# A hydrophobic groove in secretagogin allows for alternate interactions with SNAP-25 and syntaxin-4 in endocrine tissues

**DOI:** 10.1073/pnas.2309211121

**Published:** 2024-04-09

**Authors:** Edit Szodorai, Zsofia Hevesi, Ludwig Wagner, Tomas G. M. Hökfelt, Tibor Harkany, Robert Schnell

**Affiliations:** ^a^Division of Molecular and Cellular Neuroendocrinology, Department of Neuroscience, Biomedicum 7D, Karolinska Institutet, Solna SE-17165, Sweden; ^b^Department of Molecular Neurosciences, Center for Brain Research, Medical University of Vienna, Vienna A-1090, Austria; ^c^Department of Internal Medicine III, Medical University of Vienna, Vienna A-1090, Austria

**Keywords:** calcium, structural biology, synapse, vesicular exocytosis

## Abstract

Vesicular release of neurotransmitters and hormones relies on the dynamic assembly of the exocytosis/trans-SNARE complex through sequential interactions of synaptobrevins, syntaxins, and SNAP-25. Despite SNARE-mediated release being fundamental for intercellular communication in all excitable tissues, the role of auxiliary proteins modulating the import of reserve vesicles to the active zone, and thus, scaling repetitive exocytosis remains less explored. Secretagogin is a Ca^2+^-sensor protein with SNAP-25 being its only known interacting partner. SNAP-25 anchors readily releasable vesicles within the active zone, thus being instrumental for 1st phase release. However, genetic deletion of secretagogin impedes 2nd phase release instead, calling for the existence of alternative protein–protein interactions. Here, we screened the secretagogin interactome in the brain and pancreas, and found syntaxin-4 grossly overrepresented. Ca^2+^-loaded secretagogin interacted with syntaxin-4 at nanomolar affinity and 1:1 stoichiometry. Crystal structures of the protein complexes revealed a hydrophobic groove in secretagogin for the binding of syntaxin-4. This groove was also used to bind SNAP-25. In mixtures of equimolar recombinant proteins, SNAP-25 was sequestered by secretagogin in competition with syntaxin-4. K_d_ differences suggested that secretagogin could shape unidirectional vesicle movement by sequential interactions, a hypothesis supported by in vitro biological data. This mechanism could facilitate the movement of transport vesicles toward release sites, particularly in the endocrine pancreas where secretagogin, SNAP-25, and syntaxin-4 coexist in both α- and β-cells. Thus, secretagogin could modulate the pace and fidelity of vesicular hormone release by differential protein interactions.

## Significance

The precision of vesicular release, the principal form of intercellular communication in all excitable tissues, is dependent on a group of proteins that assemble in the “SNAP Receptor” (SNARE) complex at subcellular specializations. Once the content of readily available vesicles within the active zone is released, the vesicle pool and SNARE proteins need to be replenished for repetitive cycles of exocytosis. Here, we identify secretagogin as a molecular switch between syntaxin-4, a protein facilitating the movement of reserve vesicles toward the active zone, and SNAP-25, a core member of the vesicle fusion complex. Based on biological data we suggest that secretagogin can sequester either protein with its occupancy affecting the pace and magnitude of vesicular release.

The exocytosis of neurotransmitters and hormones packaged into vesicles is a fundamental mode of intercellular communication in multicellular organisms. In vertebrates, vesicular exocytosis underpins, among others, synaptic neurotransmission by fast neurotransmitters and neuropeptides in the nervous system, as well as hormone release from endocrine organs (pancreas, pituitary, adrenal gland, thyroids, and gut) (1, 2). Exocytosis takes place at subcellular specializations (synapses, release sites), allowing for the transport, fusion, reuptake, reuse, and degradation of vesicles and the associated molecular machinery. By broad definition, protein members of the *N*-ethylmaleimide-sensitive-factor attachment receptor (SNARE) family, which either localize to transmitter-laden vesicles or to the plasma membrane, interact such that a “SNARE complex” is formed to prime the fusion of vesicles at the plasmalemmal surface, followed by the liberation of its content (2–4). Vesicle fusion is precisely timed, with local Ca^2+^ entry linking excitation (e.g., action potentials, GPCR activation) to conformational changes in the proteins cooperating in the SNARE complex (5–7).

Members of the calmodulin-type Ca^2+^-sensor protein family are frontline responders to increased intracellular Ca^2+^ concentration by conformational changes in their EF-hand domains (8). This allows for the recruitment of interacting proteins to activate/inactivate downstream signaling cascades (9). Secretagogin is one such EF-hand Ca^2+^-sensor (10, 11) expressed exclusively by endocrine cells of the pancreas, adrenal medulla, pituitary, gut, stomach, and subsets of neurons in the nervous system (11–14). Secretagogin’s accumulation at the plasmalemma and on vesicles is invariable in both endocrine cells and neurons, with the abundance of secretagogin estimated as 2 to 5% of the total protein content, particularly in pancreatic β-cells (10, 11). Structural biology studies in zebrafish provided the first insights into secretagogin’s structure by defining three protein domains, each carrying two EF-hand motifs (15). Secretagogin across vertebrates differs in its N-terminal domain, leaving four EF-hand motifs with Ca^2+^-binding affinity in the low μM range (15, 16). Besides modulating the vesicular release of corticotropin-releasing hormone (CRH), insulin, glucagon, and glucagon-like peptide (11, 14, 17–20), secretagogin has also been implicated in the regulation of ionotropic receptor availability (21) and protein turnover in both developing and differentiated endocrine tissues (17). Secretagogin loss-of-function in pancreatic β-cells impairs 2nd phase insulin release, a process reliant on the trafficking of reserve vesicles, and their recruitment into the readily releasable vesicle pool (20). Despite these descriptive and mostly endocrine analyses, mechanistic and/or structural insights into how secretagogin contributes to exocytosis remain limited.

According to earlier biochemical findings using immobilized secretagogin as bait in an in vitro immunoadsorption assay (16, 22, 23), secretagogin could particularly interact with SNAP-25. Even though more recent X-ray crystallography showed that zebrafish secretagogin could indeed bind SNAP-25 (24), the direct biological demonstration of this interaction (besides putting forward the assumption that secretagogin loss-of-function ought to require cytosolic partner(s) to impair synapse formation and integrity) (24), and if any such interaction is indeed exclusive to SNAP-25, remain unresolved. Accordingly, secretagogin’s interaction partners, the role(s) of any such protein–protein interaction, and its cell type-specificity in mammals are unknown.

Here, we describe the focused screening of secretagogin binding partners among proteins that participate in the SNARE machinery. By filtering invariable hits in proteomics-based screens in endocrine cells and neurons (10, 16–18, 22, 23), both syntaxin-1A and syntaxin-4 have been identified in addition to SNAP-25. We characterized α- and β-cells of the endocrine pancreas where secretagogin coexists with both SNAP-25 and syntaxin-4. We then showed high-affinity Ca^2+^-dependent direct interactions between secretagogin and both syntaxin-4 and SNAP-25, identified their interaction target sites, solved the X-ray structures of both mouse and human protein-peptide complexes, and analyzed the effect of single amino acid (AA) substitutions to highlight residues critical for ligand binding. We also found that secretagogin preferred SNAP-25 when both ligands are equimolarly present, a finding that led us to manipulate syntaxin-4 levels in cell systems and link their changes to altered insulin release. We concluded that secretagogin could act as a chaperon to scale the availability of SNARE proteins in situ, with its binding to syntaxin-4 facilitating the recruitment of vesicles from the reserve pool for exocytosis. Secretagogin’s potential partner switching, if occurs sequentially in vivo, could represent a dual action for a Ca^2+^-sensor protein.

## Results

### Secretagogin Binding Domains in SNAP-25 and Syntaxin-4.

**Fig. 1.**
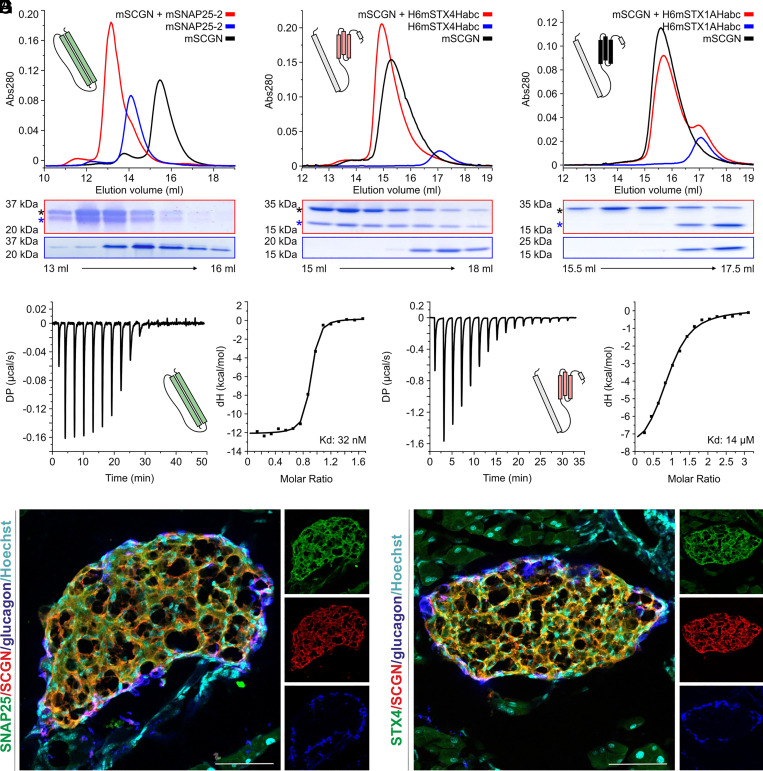
Secretagogin binds both SNAP-25 and syntaxin-4. Profiling of pair-wise interactions by size exclusion chromatography between mouse secretagogin and isoform-2 of SNAP-25 (*A*), the H_abc_ module of syntaxin-4 (*B*), and the H_abc_ module of syntaxin-1A (*C*). SDS gels show the peak fractions under each chromatogram. Asterisks label the individual proteins. (*D*, *Left*) Secretagogin (mouse) binding to SNAP-25 (isoform-2) as determined by ITC-based analysis, with the calculated K_d_ = 32 nM (*Right*). (*E*) Secretagogin (mouse) binding to the H_abc_ module of syntaxin-4 (ITC analysis; binding curve to the *Left*), with K_d_ = 14 µM (*Right*). (*F* and *G*) Histochemical localization of secretagogin (SCGN), SNAP-25 (*F*) or syntaxin-4 (*F*) in the endocrine pancreas of mice. Given the core-shell distribution of β- and α-cells in mammalian pancreatic islets, glucagon was used to mark α-cells with the bulk of core cells being β-cells (17, 18). Hoechst 33,421 was used as nuclear counterstain. *Insets* to the right show channel separation. (Scale bars, 60 μm.)

Secretagogin pull-down in endocrine cells and neurons (18, 22) revealed several constituents of the SNARE complex (*SI Appendix*, Fig. S1*A*) with similar probability as SNAP-25. Therefore, we asked whether core SNARE component(s) other than SNAP-25 could directly interact with secretagogin in a Ca^2+^-dependent fashion. To this end, recombinant mouse secretagogin was produced and purified along with the individual modules and domains of SNARE proteins (*SI Appendix*, Fig. S1*A*, for constructs, see Dataset S1). First, we used size exclusion chromatography to probe the pairwise binding of secretagogin to VAMP2, VAMP8, syntaxin-1A, syntaxin-4 along with SNAP-25 in the presence of Ca^2+^. We confirmed that secretagogin binds both SNAP-25 isoforms (see *SI Appendix*, Fig. S2*A* and Fig. 1*A* for SNAP25-1 and SNAP25-2, respectively). Secretagogin did not bind either VAMP2 or VAMP8 (*SI Appendix*, Fig. S2 *B* and *C*). To test an interaction between syntaxin-1/-4 and secretagogin, we used the individual H_abc_- and SNARE-domains of both syntaxin-1A/-4 because they can exist in both open and closed conformations (*SI Appendix*, Fig. S1 *B* and *C*) (25–27). Secretagogin interacted with the globular H_abc_ domain of syntaxin-4 (Fig. 1*B*), but not its SNARE domain (*SI Appendix*, Fig. S2*D*). In contrast, it failed to show any interaction with either the SNARE or the H_abc_ domain of syntaxin-1A (Fig. 1*C* and *SI Appendix*, Fig. S2*E*). These data identified syntaxin-4 as a binding partner for secretagogin.

We then used isothermal titration calorimetry (ITC) to validate the Ca^2+^-dependence and stoichiometry of secretagogin-target interactions. For secretagogin and SNAP25-2, K_d_ was ~32 nM (Fig. 1*D* and *SI Appendix*, Fig. S2*G* and Table S1). Similarly, the syntaxin-4-derived H_abc_ module was verified as a Ca^2+^-dependent partner, with K_d_ = ~14 µM, and 1:1 stoichiometry (Fig. 1*E* and *SI Appendix*, Fig. S2*H*). Based on K_d_ comparisons, we suggest that secretagogin might prefer SNAP-25 over syntaxin-4, at least in recombinant systems.

### Colocalization of Secretagogin, SNAP-25, and Syntaxin-4 in Pancreatic Islets.

Secretagogin was discovered as a marker of insulin-producing β-cells in the endocrine pancreas (11). Thus, we asked whether secretagogin could coexist with both SNAP-25 and syntaxin-4 in the islands of Langerhans. Our hypothesis was supported by proteomic (16, 18), bulk- and single-cell RNA-seq data (for secretagogin: https://www.proteinatlas.org/ENSG00000079689-SCGN/tissue; for SNAP-25: https://www.proteinatlas.org/ENSG00000132639-SNAP25/; and for syntaxin-4: https://www.proteinatlas.org/ENSG00000103496-STX4/tissue) (28, 29) on the cellular distribution of the protein targets. By using multiple-label histochemistry we found secretagogin, SNAP-25, and syntaxin-4 to colocalize in both α-cells (glucagon^+^ in the shell compartment of islands) and β-cells (in the core of the islets; Fig. 1 *F* and *G*). The anatomical data were confirmed by qPCR detection of the three transcripts in both INS-1E [rat insulinoma; (ΔCT values: 15.2 (*Gapdh*), 20.6 (*Stx4*), 20.2 (*Snap25*), and 17.1 (*Scgn*)) and αTC1-6 cell lines (mouse adenoma; ΔCT values: 14.3 (*Gapdh*), 31.1 (*Stx4*), 17.9 (*Snap25*), and 28.0 (*Scgn*)]. These data highlight the potential significance of secretagogin-syntaxin-4 interaction in the physiology of hormone release.

### Mapping Secretagogin’s Target Site in the C-terminal Helix of SNAP-25.

**Fig. 2.**
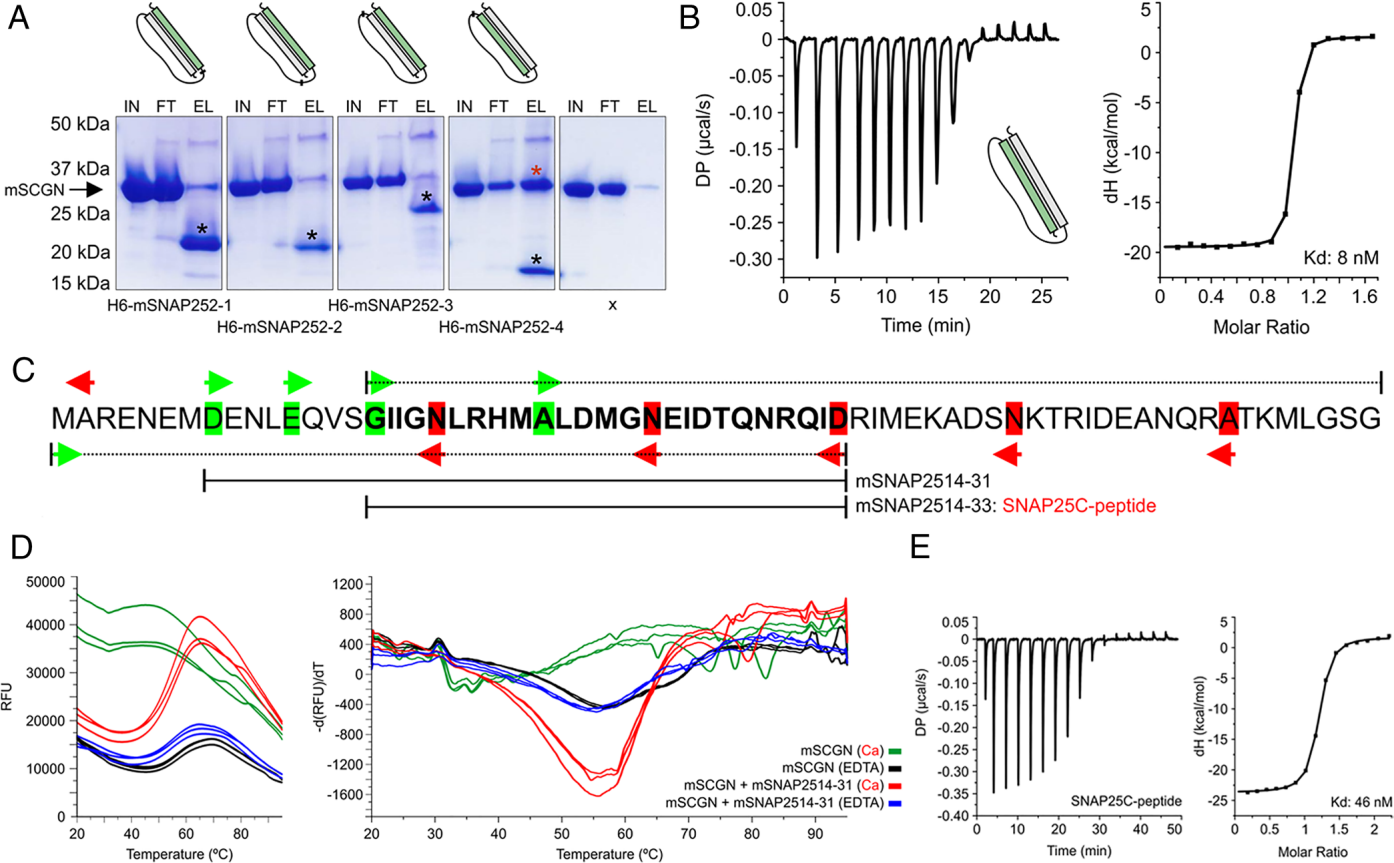
Identification of the target motif for secretagogin in SNAP-25. (*A*) Pull-down assay using four His-tagged fragments derived from mouse SNAP-25 isoform-2 (designated as mSNAP25-n) as bait implicated the C-terminal helix of SNAP-25 in secretagogin binding. Asterisks in black identify the SNAP-25 fragments. Asterisk in red denotes secretagogin (mouse; mSCGN). (*B*) K_d_ = 8 nM was calculated for the interaction between mSCGN and the C-terminal helix of mouse SNAP-25 by ITC. (*C*) Schema of N- and C-terminal polar deletions in the C-terminal helix of SNAP-25. Arrows in green and red point to start and stop positions, respectively. Dotted and solid lines above and underneath the protein sequence identify the shortest constructs, SNAP-25^14–31^ and SNAP-25^14–33^, that retained mSCGN binding. (*D*) Differential scanning fluorimetry plots of mSCGN with the SNAP-25^14–31^ peptide ligand (triplicates). Both the denaturation kinetics (*Left*) and their derivatives (−dF/dT, *Right*) were shown. (*E*) Binding curve (*Left*) and derived K_d_ (46 nM; *Right*) for the interaction between mSCGN and mSNAP-25^14−33^, as revealed by ITC.

To map the target site in mouse SNAP-25, we generated recombinant constructs comprising its individual α-helical segments and used these as His_6_-tagged baits in pull-down experiments. Only the C-terminal SNARE domain (SNAP-25c), which has identical sequence in both the SNAP25-1 and -2 isoforms, exhibited secretagogin binding (Fig. 2*A* and *SI Appendix*, Fig. S2 *F* and *I*) with 1:1 stoichiometry and K_d_ = ~8 nM in the presence of Ca^2+^ (Fig. 2*B*). EDTA, a Ca^2+^ chelating agent, prevented SNAP-25c binding (*SI Appendix*, Fig. S2*J*).

Next, we used N- and C-terminal polar deletions of SNAP-25c to engineer eight constructs (Fig. 2*C* and Dataset S1) to map the secretagogin binding site precisely within its long α-helix (*SI Appendix*, Fig. S3*A*). We have identified the 155-GIIGNL RHMALDMGNEIDTQNRQID-179 peptide stretch as the shortest (25 AA-long) α-helical segment to retain secretagogin binding and confirmed this interaction by both differential scanning fluorimetry (Fig. 2*D*) and ITC (K_d_ = ~46.6 nM; Fig. 2*E*).

Differential scanning fluorimetry indicated that the SNAP-25c motif 147-MADENLEQVSGIIGNLRHMALDMGNEIDTQN RQID-179 increases fold stability and likely occupies the exposed hydrophobic pocket formed within secretagogin upon Ca^2+^ binding. The docking of this SNAP-25c-derived peptide on secretagogin produced a signal reminiscent of globular folded proteins (Fig. 2*D*). These data allowed us to generate a fusion protein for crystallization by attaching this SNAP-25c motif (AA155-179) to the C-terminus of green fluorescent protein (GFP). We then confirmed this chimeric GFP-linked SNAP-25-peptide to retain secretagogin binding when coexpressed and copurified in a complex (*SI Appendix*, Fig. S3*B*). Since GFP carrying the SNAP-derived peptide (155-GIIGNLRHMALDMGNEIDTQNRQ ID-179) at its C-terminus allowed for the formation of a stable complex with secretagogin, this ensemble was used in X-ray crystallography studies.

### Both Mouse and Human Secretagogin Bind SNAP-25c.

The mouse secretagogin sequence shares 85% identity with the corresponding human protein (Dataset S1). Thus, we sought to address if human secretagogin also interacts with SNAP-25c. When testing for pairwise interactions by coexpression, copurification, and size-exclusion chromatography, recombinant human secretagogin also bound the GFP-SNAP-25c chimera in the presence of Ca^2+^ (noting that mouse and human SNAP-25c are identical). Thus, recombinant human secretagogin together with the GFP-fused SNAP25-derived peptide could also be used in X-ray crystallization aimed at resolving the structure of ligand-bound human secretagogin.

### Crystal Structure of Secretagogin in Complex with a SNAP-25-Derived Ligand.

**Fig. 3.**
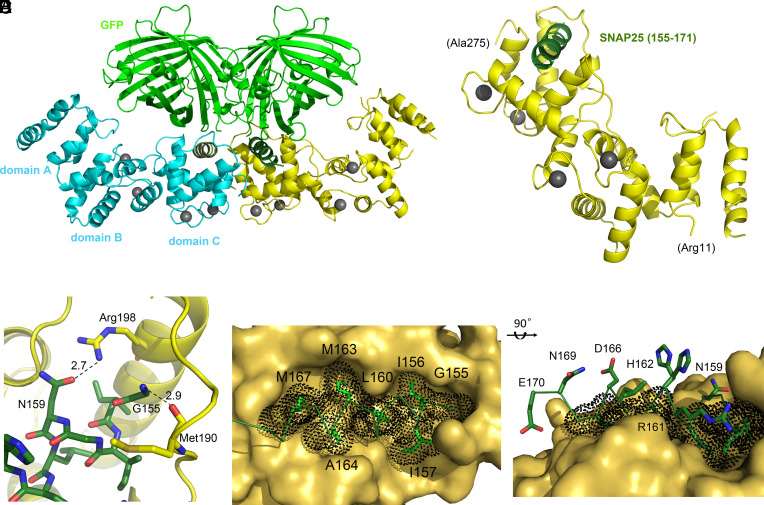
Secretagogin in complex with a SNAP-25-derived peptide. (*A*) Crystal structure of secretagogin (mouse; mSCGN in cyan and yellow) complexed with a GFP-SNAP25-derived peptide (light and dark green, respectively). Two copies of the complex, corresponding to the asymmetric unit of the P2_1_2_1_2_1_ crystal, were depicted as cartoons. Ca^2+^ ions were shown as gray spheres. (*B*) mSCGN (yellow) with the bound helical SNAP-25-peptide (dark green). Ca^2+^ ions appear as gray spheres. (*C*) Hydrogen bonds formed between mSCGN (yellow carbons), and its peptide ligand (green carbons) shown as dashed lines with their distances given in Å. (*D*) Hydrophobic contact area of the SNAP25-derived peptide (sticks in green) filled the binding groove (surface). Dot-surface represents Van der Waals radii. (*E*) Side view of the bound peptide ligand in the binding groove of mSCGN with the polar side-chains on the surface.

We determined the X-ray structure of both mouse and human secretagogin bound to the SNAP-25-derived peptide ligand at 2.35 Å and 2.30 Å resolution, respectively (Fig. 3 and *SI Appendix*, Fig. S4). In both cases, GFP carrying the target peptide (155-GIIGNLRHMALDMGNEIDTQNRQID-179) as C-terminal tag was employed whereby the large globular GFP module served as a crystallization chaperon that was necessary to obtain diffracting crystals (see *SI Appendix*, Table S2 for statistics and model parameters).

Secretagogin is built by three domains (denoted here as A, B and C), each comprising a pair of EF-hand motifs (*SI Appendix*, Fig. S1*C*). Both mouse and human secretagogin exhibit 4 Ca^2+^-binding sites in their B and C domains. In contrast, the N-terminal A domain lacks Ca^2+^ ions consistent with the sequence alterations in both EF-hand motifs (15, 16). The P2_1_2_1_2_1_ crystal’s asymmetric unit contained two copies of the protein complex (Fig. 3*A*), wherein the secretagogin molecules were identical; superposition results in a Cα-rmsd of 0.54 Å and 0.66 Å for the mouse and the human proteins, respectively. Even for GFP-mediated crystal contacts, its interaction with the SNAP-25c fragment was limited to 1 and 2 hydrogen bonds for mouse and human structures, respectively, making the target peptide freely available to secretagogin. The SNAP-25-derived peptide was bound at the groove formed between the two EF-hand modules of the C domain of secretagogin (Fig. 3*B*).

The electron density map (*SI Appendix*, Fig. S5*A*) allowed us to model a 17 AA-residue (155-GIIGNLRHMALDMGNEI-171) and a 16 AA-residue (155-GIIGNLRHMALDMGNE-170) section of the SNAP-25 peptide for the mouse and human secretagogin complexes, respectively. The remaining 8 (172-DTQNRQID-179) and 9 (171-IDTQNRQID-179) residues lacked electron density and appeared disordered. These sections were situated outside the binding groove, did not interact with secretagogin, and had no other crystal contact. Contact points between secretagogin and the SNAP-25-peptide were dominated by hydrophobic interactions (155-*G***II**G*N***L**RH**MAL**D**MG** NEI-171, *polar interaction*/**hydrophobic-stacking**). The sporadic polar contacts were hydrogen bonds (Fig. 3*C*) present in both copies of the asymmetric unit between i) the backbone amine of G155 and the carbonyl of Met190 (distance 2.7 Å and 2.9 Å, respectively) and ii) the side chain amide of N159 and Arg198 (distance 2.9 Å and 2.7 Å, respectively). Hydrophobic interactions (Fig. 3 *D* and *E*) extended on the buried interface of SNAP-25c (155-GIIGNLRHMALDM-167), particularly at residues I156, I157, L160, M163, A164, M167 in the groove formed by the Ca^2+^-loaded C domain of secretagogin, presenting Van der Waals distances between 3.5 and 3.9 Å to the surrounding residues.

The above interactions were replicated in the human secretagogin complex (Fig. 3 and *SI Appendix*, Fig. S4), in both copies of its asymmetric unit. The buried surface area of the peptide ligand in the mouse secretagogin was measured as 914 Å^2^, corresponding to 51.7% of the total surface area (1,765 Å^2^) of the 16 AA-long SNAP-25c segment within the complex. We obtained similar values for human secretagogin with a buried surface area of 886 Å^2^, accounting for 50.9% of the total surface area (1,740 Å^2^). The size and proportion of such hydrophobic contact surfaces support the affinity of the interaction measured by ITC for these peptide segments.

### Mapping Secretagogin’s Target Motif within the H_abc_ Domain of Syntaxin-4.

**Fig. 4.**
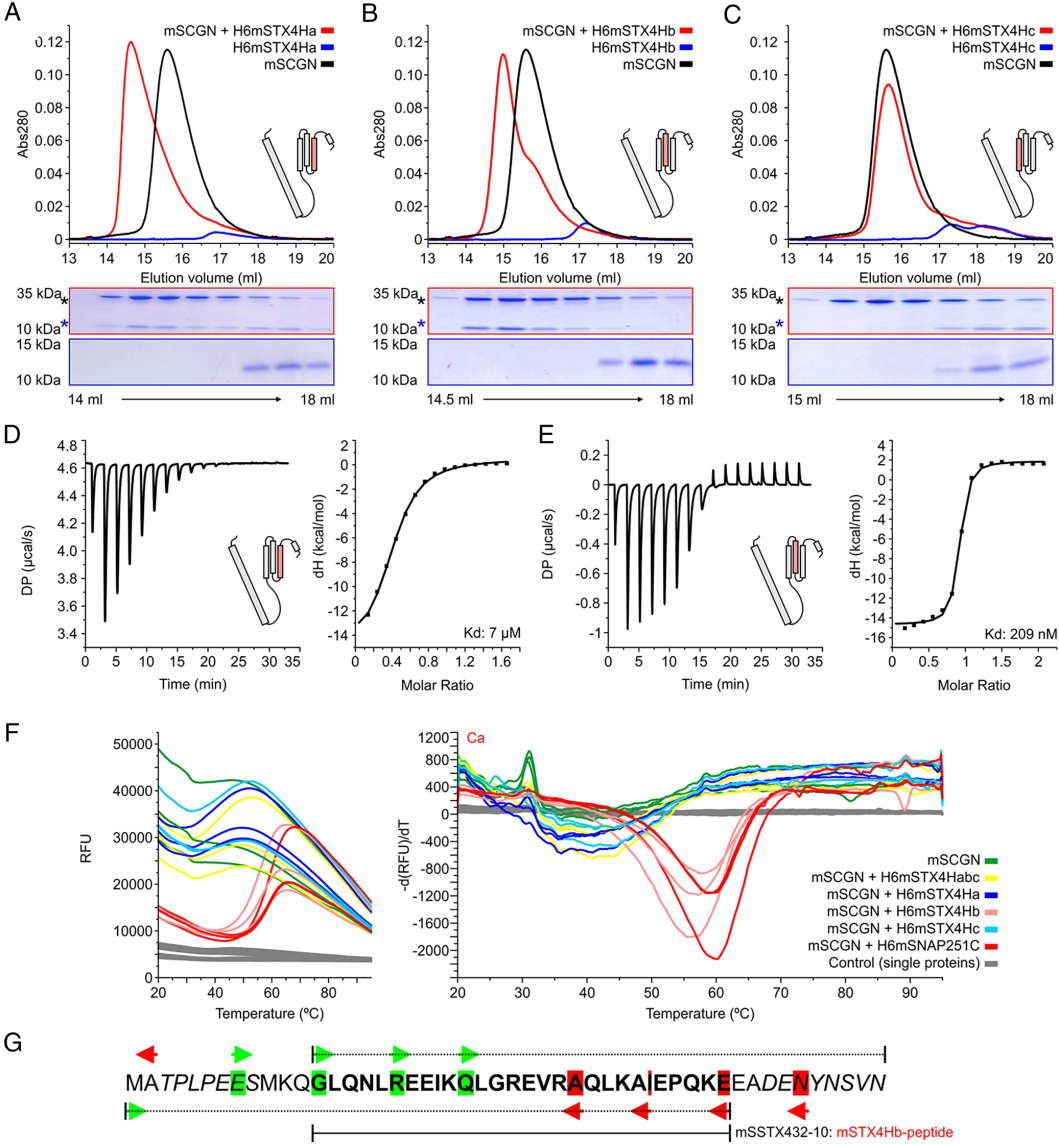
The target site of mouse secretagogin in the H_abc_ domain of syntaxin-4. (*A*–*C*) Elution profiles (size exclusion chromatography) of for mouse secretagogin (mSCGN) in the presence of the individual helices of the H_abc_ domain: helix-a (H_a_, *A*), helix-b (H_b_, *B*) and helix-c (H_c_, *C*). (*D* and *E*) ITC recordings of secretagogin binding to H_a_ (*D*) and H_b_ (*E*). The binding curve (*Left*) and calculated K_d_-s (7 µM for H_a_; 209 nM for H_b_; *Right*) were shown. (*F*) Denaturation kinetics of mSCGN with the individual helices of syntaxin-4 (H_a_, H_b_, H_c_; *Left*) and their derivatives (−dF/dT; *Right*) shown by differential scanning fluorimetry (triplicates). H6mSNAP251C, a SNAP-25-derived peptide ligand, served as positive control. Data recorded with the individual helical constructs (H_a_, H_b_ and H_c_; single proteins) were color-coded in gray. (*G*) N- and C-terminal polar deletion constructs of the H_b_ helix, with start and stop positions in green and red, respectively. The shortest construct that retained binding to mSCGN (mSTX4^32–10^, also termed mSTX4Hb-peptide) was outlined by dotted and solid lines.

The identification of the H_abc_ domain of syntaxin-4 as a Ca^2+^-dependent binding partner for secretagogin (Fig. 1 *B* and *E*) prompted us to define the structural basis of their interaction. Therefore, we generated constructs corresponding to the three individual helices of the H_abc_ domain (termed Ha, Hb, and Hc) with overlapping sequences of their interconnecting loops (Fig. 4 *A*–*C* and Dataset S1). When using these constructs in size exclusion chromatography (Fig. 4 *A*–*C*) and pull-down assays as bait (*SI Appendix*, Fig. S3*C*), we found that the Ha and Hb helices retained secretagogin binding. Biophysical analysis by ITC revealed K_d_ = ~209 nM and K_d_ = ~7 µM for Hb and Ha, respectively (Fig. 4 *D* and *E*), pointing at Hb as the high affinity ligand. Differential scanning fluorimetry indicated that syntaxin-4 Hb occupied the exposed hydrophobic pocket formed within secretagogin upon Ca^2+^ binding, analogous to the SNAP-25-derived ligand (Fig. 4*F*).

Subsequently, N- and C-terminal polar deletions of the syntaxin-4 Hb helix were undertaken to map the exact target site. Short His_6_-tagged peptide constructs (Fig. 4*G*) served as bait in a pull-down assay (*SI Appendix*, Fig. S3*D* and Dataset S1). GFP-fusion constructs carried these peptides as C-terminal tags for biophysical characterization and crystallization trials. A 28 AA-long peptide (82-GLQNLREEIKQLGREVRAQLKAIEP QKE-109; as GFP-fusion tag termed GFPmSTX4Hb10) was the shortest segment that still exhibited nM binding (K_d_ = 826 nM, *SI Appendix*, Fig. S3*E*). Improved affinities of 275 nM and 541 nM were measured for longer constructs 72-TPLPEESMKQ GLQNLREEIKQLGREVRAQLKAIEPQKE-109 (GFPmSTX4Hb7) and 77-ESMKQGLQNLREEIKQLGREVRA QLKAIEPQKE-109 (GFPmSTX4Hb9) (*SI Appendix*, Table S1). These data suggest that a 28 AA-long helical stretch in the H_abc_ domain of syntaxin-4 dictates its high-affinity interactions with secretagogin.

Next, we addressed the domains of mouse secretagogin that serve as interaction site(s) for syntaxin-4, for which the A+B, B, B+C, and C domains of secretagogin were recombinantly produced (Dataset S1). When using size exclusion chromatography and pull-down assays, we found that both its B and C domains were required for optimal binding of the H_abc_ domain of syntaxin-4 (even though the C domain alone retained interaction; *SI Appendix*, Fig. S6). Coincidently, the B domain of secretagogin provides auxiliary support for fold stabilization.

### Both Mouse and Human Secretagogin Bind Syntaxin-4.

We tested binding of human secretagogin to the H_abc_ domain, and specifically to the Hb helix, of human syntaxin-4 (Dataset S1). Size-exclusion chromatography revealed that human secretagogin and human syntaxin-4 equally formed complexes in the presence of Ca^2+^ (*SI Appendix*, Fig. S7). These results highlight the potential presence of a secretagogin-syntaxin-4 interaction in all mammals.

### Crystal Structure of Secretagogin in Complex with a Syntaxin-4-Derived Target Peptide.

**Fig. 5.**
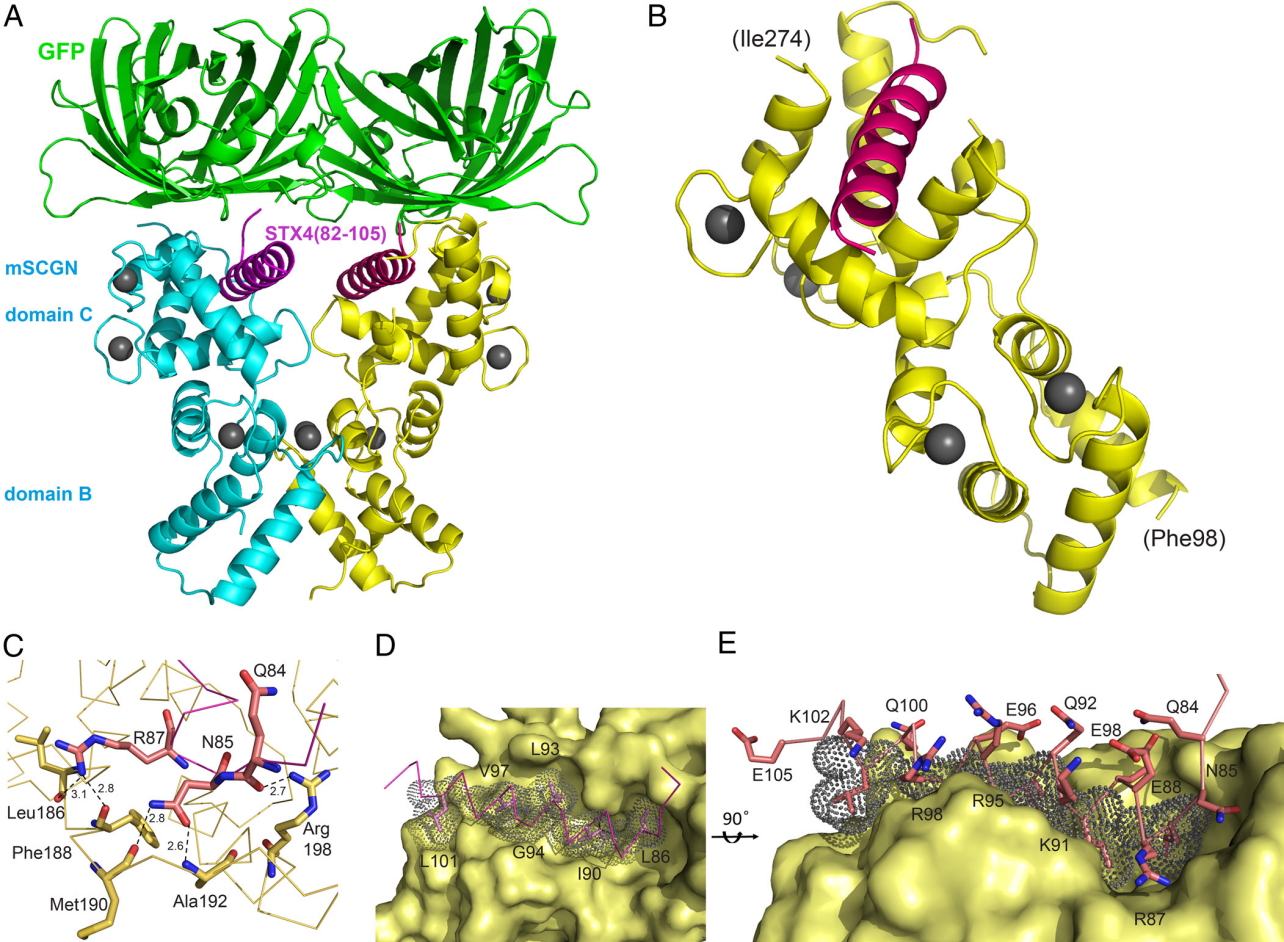
Secretagogin in complex with the syntaxin-4-derived peptide. (*A*) Cocrystal structure of secretagogin (mouse; mSCGN, cyan and yellow) and a chimeric GFP-syntaxin-4 (STX4)-derived peptide. Cartoon shows two copies of a 1:1 complex at the asymmetric unit of the P2_1_2_1_2_1_ crystal. GFP chains (green) were fused with syntaxin-4-derived peptide (purple). (*B*) Two-domain module of mSCGN (yellow) with bound syntaxin-4 peptide (purple). Ca^2+^ ions in *A* and *B* were depicted as gray spheres. (*C*) Dashed lines correspond to hydrogen bonds formed between mSCGN (beige carbons) and the peptide ligand (pink carbons), with distances given in Å. (*D*) The hydrophobic contact area is in the binding groove of mSCGN with syntaxin-4 peptide residues (in pink) depicted as sticks. Dot-surface represents Van der Waals radii. (*E*) Side-view of the bound peptide ligand within the binding groove with polar side chains of the bound syntaxin-4 peptide located on the surface.

We pursued crystallization trials with the BC-module (residue range AA90-276) of mouse secretagogin, noting that the flexible, Ca^2+^ free N-terminal domain presented higher B-factors in two independent crystal structures (*SI Appendix*, Fig. S5*B*), and was not part of its binding site. We presented the 82-GLQNLREEIKQLGREVRAQLKAIEPQKE-109 syntaxin-4-derived peptide as a C-terminal tag on GFP for secretagogin binding (Dataset S1). Secretagogin/GFP-syntaxin-4-peptide complexes were isolated by preparative size exclusion chromatography. Diffracting crystals were obtained with the structure of the protein complex determined at 2.65 Å resolution (for statistics and model parameters see *SI Appendix*, Table S2). The crystals belonged to the P2_1_2_1_2_1_ space group; yet were distinct from the earlier secretagogin-SNAP-25 complex in their packing and unit cell dimensions. The asymmetric unit contained two copies of the protein complexes (Fig. 5*A*). The two secretagogin chains were nearly identical, with superposition resulting in a Cα-rmsd of 0.72 Å based on 177 aligned residues. GFP, acting as crystallization chaperon, mediated crystal contacts predominantly between GFP molecules. Contacts between GFP and the syntaxin-4-derived peptide were limited to one and two H-bonds in the two copies. The syntaxin-4-derived peptide ligand was bound at the C-domain of secretagogin in the same groove as the SNAP-25-derived peptide (Fig. 5*B*).

The electron density map (*SI Appendix*, Fig. S5*A*) allowed the modeling of a 22 AA (Q84-E105) and a 24 AA (G82-E105) segment of the 28 AA-long syntaxin-4 peptide stretch in the respective copies of the asymmetric unit. In the linker region between the GFP and the syntaxin-4 fragment, peptide flexibility resulted in disorder; one and three residues could not be modeled in the respective chains. Four residues at the C-terminus (106-PQKE-109) extended beyond the binding groove, lacked contacts and electron density, and hence could not be modeled either. Here, we based the structure description on a complex, which displayed lower B-factors. The syntaxin-4 fragment was bound at the groove formed by the Ca^2+^-loaded EF-hand modules within the C domain of secretagogin, involving each of the five helices (Fig. 5*B*). The binding was stabilized by polar contacts and hydrogen bonds at the N-terminal end, whereas the remaining segment was dominated by hydrophobic stacking between apolar sidechains (82-GL*QN***L***R*E*E***I**KQ**LG**RE**V**RAQ**L**KAIEPQKE-109 *polar interaction*/**HF-stacking**). Peptide residues Q84, N85, and R87 formed hydrogen bonds with the side chain of Arg198, and the backbone atoms of Ala192, Met190, Phe188, and Leu186 from secretagogin (Fig. 5*C*). The segment, which formed hydrophobic interactions included contact residues L86, I90, L93, G94, V97, and L101 with Van der Waals distances in the range of 3.5 to 4.0 Å to the residues building the groove (Fig. 5 *D* and *E*). The above interactions were replicated in both copies of the crystal asymmetric unit. The buried surface area of the peptide was 1,028 Å^2^ and 1,068 Å^2^ in the two copies, corresponding to 43.4% and 40.4% of the total surface area of the modeled 24 AA-long syntaxin-4 fragment.

### Comparison of Secretagogin Complexes Harboring SNAP-25 or Syntaxin-4 Derived Peptides.

**Fig. 6.**
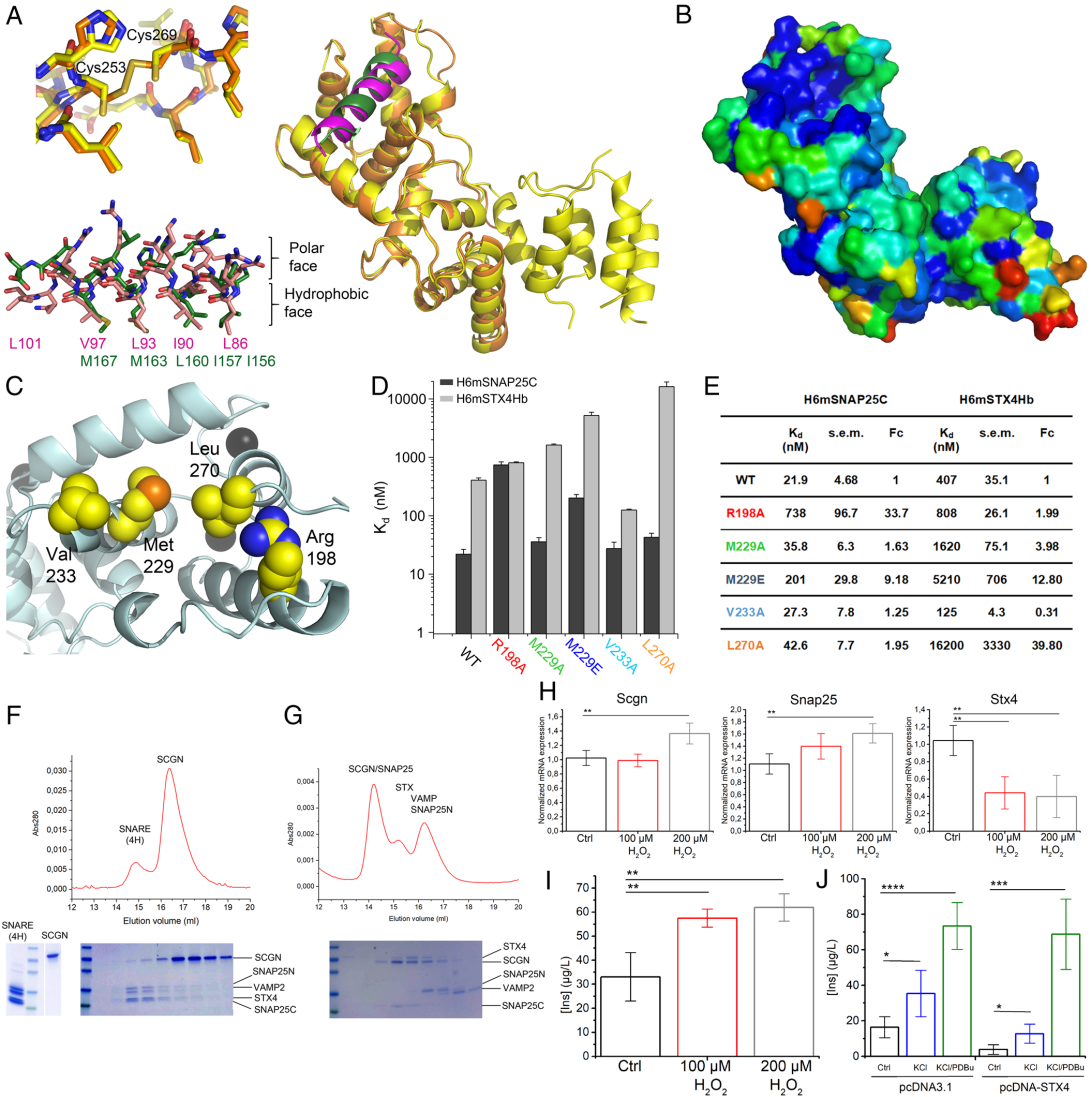
Comparing the ligand-bound states of secretagogin. (*A*) Superposition of secretagogin (mouse; mSCGN) complexes with SNAP-25-derived (yellow, dark green) and syntaxin-4-derived peptides (orange, purple). *Insets* show disulfide bonds in the syntaxin-4-peptide complex. Peptide ligands were drawn as sticks with their residues forming the hydrophobic contact (*Bottom Left*). (*B*) Residue conservation on the surface of mSCGN based on 24 SCGN sequences, with orientation as in *A*. In a rainbow spectrum, dark blue and red represent the most conserved and most variable positions, respectively. (*C*) Highlighted residues in the binding grove of mSCGN (cyan cartoon) were selected for mutagenesis. (*D*) K_d_-s of mSNAP-25-derived peptide (black) and syntaxin-4-derived peptide (gray) recorded with both wild-type (WT) and mutant mSCGN. Data in *D* were expressed as means ± SEM. (*E*) ITC-based affinity data for the two peptide ligands and mSCGN mutants listing the K_d_, SEM (s.e.m.), and fold change (Fc) relative to those obtained WT mSCGN. (*F*) Preformed SNARE complex including four SNARE helices (4H: SNAP-25N, SNAP-25C, syntaxin-4 and VAMP2), were used to challenge mouse secretagogin (mSCGN). Size exclusion chromatography revealed two peaks in the elution profile. Representative SDS gels are shown under the chromatograms. Mouse SCGN did not bind the C-terminal helix of SNAP-25 when it is incorporated in a SNARE complex. (*G*) Preformed complexes of mSCGN and the syntaxin-4 (H_abc_ and SNARE domains in open conformation) were added to the SNARE helices (SNAP-25N, SNAP-25C, and VAMP2). Size exclusion chromatography, resulted in several peaks in the elution profile indicating that mSCGN preferentially binds the C-terminal helix of SNAP-25 (SNAP-25C), while the remaining proteins migrated separately. (*H*) Normalized mRNA expression levels in response to H_2_O_2_ (triplicates). (*I*) Insulin release by INS-1E cells was increased by H_2_O_2_. (*J*) Syntaxin-4 overexpression reduced both basal (*P* = 0.017; control vs. overexpression) and KCl-induced (*P* = 0.033; control vs. overexpression) insulin release. In contrast, PDBu evoked maximal insulin release regardless of the genetic manipulation ((*P* = 0.712; control vs. overexpression; all in quadruplicate). Data were expressed as means ± SEM; *P < 0.05, **P < 0.01, ***P < 0.001.

The BC module of secretagogin in the syntaxin-4-peptide complex was identical to the structure of the corresponding segment of the three-domain secretagogin in the SNAP-25c-peptide complex. Structural superposition based on 177 aligned residues returned a Cα-rmsd of 0.8648 Å (Fig. 6*A*). The four Ca^2+^-binding sites were retained in the structures (Figs. 3*B* and 5*B*). Both the SNAP-25 and the syntaxin-4-derived peptides were bound at the same groove, aligned in the same helical phase utilizing the pockets for docking the hydrophobic side chains (Fig. 6*A*), while hydrophilic residues remained exposed on the surface. The buried interaction areas accounted for 886 to 1,068 Å^2^, equivalent to 40 to 50% of the surface area, in both complexes. One apparent difference was the presence of a disulfide bond between C253 and C269 of secretagogin in both chains of the asymmetric unit of the syntaxin-4-peptide-bound complex. This was absent in the SNAP-25-peptide-bound structure. This disulfide bond, however, affected neither the relative orientation of the EF-hand domains nor the conformation of the surrounding AA sidechains (Fig. 6*A*). Residue conservation based on 24 sequences from vertebrates (including fish, amphibians, reptiles, and mammals) mapped in three dimensions confirmed this binding groove be the most evolutionarily conserved area in secretagogin (Fig. 6*B*).

### Mutations in the Binding Groove of Secretagogin Disrupt Peptide Interactions.

We have identified the interactions and AA residues that served as contact points in the complexes of both mouse and human secretagogin. Next, we designed Ala replacements at positions Arg198 (R198A), Met229 (M229A), Val233 (V233A), and Leu270 (L270A) in mouse secretagogin (Fig. 6*C*). Since the interacting peptides are embedded in a large and predominantly hydrophobic binding groove, single Ala replacements might be ineffective to impair binding. Therefore, an additional mutant with the hydrophobic side chain of Met229 changed to glutamate (M229E) was produced (for constructs see Dataset S1). ITC (Fig. 6 *D* and *E* and *SI Appendix*, Fig. S8 *A* and *B* and Table S1), and differential scanning fluorimetry (*SI Appendix*, Fig. S8 *C–F*) were employed to characterize the interaction of these mutants with the SNAP-25c and syntaxin-4-derived Hb fragments. The mutations affected neither folding integrity nor thermal stability, as derived from the thermal denaturation kinetics in the presence/absence of Ca^2+^ (*SI Appendix*, Fig. S8 *E* and *F*). The R198A mutation impaired secretagogin binding to SNAP-25c. The M229A mutation had a remarkably smaller effect. M229E replacement significantly impeded the binding of both target peptides. V233A had a marginal effect (noting that it left most of the contact area intact). L270A only impaired secretagogin binding to the syntaxin-4-derived Hb fragment. These results suggest a pivotal role of the H-bond by Arg198, and the central part of the hydrophobic groove for both binding partners, with Leu270 specifically affecting the syntaxin-4-derived peptide. Nevertheless, the affinity data and the analysis of these mutants validated the binding mode described by the X-ray structures (Fig. 6*A*).

### Secretagogin Binds Both the Open and Closed Conformations of Syntaxin-4 Monomers.

Syntaxin-4 exists in both open and closed conformations (*SI Appendix*, Fig. S9*A*), with their transition rooted in the relative movement of its H_abc_ and SNARE modules. The wild-type sequence represents the closed conformation (27). In contrast, mutations in the interdomain hinge between the H_abc_ and SNARE domains lock syntaxin-4 in its open conformation (27). Here, we used both wild-type syntaxin-4 and a L173A/E174A double mutant (for constructs see Dataset S1) to assess the conformation dependence of secretagogin binding. Simultaneously, we tested the effect of the 31 N-terminal residues (termed “N-peptide”), which is known to regulate syntaxin-4 interactions (30, 31). Size exclusion chromatography showed that the N-peptide hinders syntaxin-4 binding to secretagogin regardless of it being the two domain construct or the isolated H_abc_ domain (*SI Appendix*, Fig. S9 *B* and *D*). L173A/E174A substitutions had no impact on this interaction (*SI Appendix*, Fig. S9 *B, C, E,* and *F*) indicating that syntaxin-4 in both open/closed conformation can bind secretagogin. Pull-down experiments using His_6_-tagged syntaxin-4 with/without the N-peptide demonstrated that the presence of the N-peptide weakened its binding to secretagogin (*SI Appendix*, Fig. S9*G*).

Syntaxin-4 is prone to oligomerize when purified at high concentrations in vitro (*SI Appendix*, Fig. S9). Secretagogin only binds syntaxin-4 monomers. We used mass photometry (32), which allows the detection of proteins at concentrations 1,000-fold lower than those used in size exclusion chromatography; with the limitation that proteins <50 kDa cannot be detected. By using recombinant syntaxin-4 that lacked the N-peptide, we confirmed its propensity to form oligomers (*SI Appendix*, Fig. S10*A*). Moreover, we confirmed that secretagogin only binds syntaxin-4 monomers (*SI Appendix*, Fig. S10 *B* and *C*). Overall, data from ITC, size exclusion chromatography, X-ray structure determination, and mass photometry collectively suggest that the Hb helix in the H_abc_ domain of syntaxin-4 is a bona fide binding partner for secretagogin.

### Secretagogin Affects SNARE Assembly In Vitro.

An equimolar mixture of the SNARE domains of recombinant syntaxin-4, SNAP-25 and VAMP2 (Dataset S1 for constructs, all from mouse) was produced to mimic a minimal preformed four-helix SNARE complex in vitro. Size exclusion chromatography was used to show that secretagogin does not bind to SNAP-25c when it is embedded in the SNARE complex (Fig. 6*F*). Next, we asked whether the difference in affinity for SNAP-25c and syntaxin-4 by secretagogin (K_d_ = 32 nM and K_d_ = 14 μM, respectively) could be reflected in preference for partner recruitment. To this end, we isolated secretagogin/full-length syntaxin-4 complexes and mixed these with the SNARE-domains of SNAP-25 and VAMP2 (at equimolar concentrations). Size exclusion chromatography showed that secretagogin-bound syntaxin-4 was replaced by SNAP-25c, the high-affinity ligand (Fig. 6*G*).

### Quality Control of Antibodies for the Localization of Protein Complexes.

The tissue-resolved anatomy data presented in Fig. 1 *F* and *G* could be biased if the antibodies used recognize epitopes that are inaccessible due to the formation of protein complexes. Therefore, we have performed epitope mapping for secretagogin, SNAP-25 (*SI Appendix*, Fig. S11*A*) and syntaxin-4 (*SI Appendix*, Fig. S11*B*). These data show that all antibodies bind freely accessible protein epitopes, and complex formation could per se not interfere with target visualization in vivo. This information also allowed us to perform localization studies at single-vesicle resolution, both showing the coexistence of target proteins in α- and β-cells (*SI Appendix*, Fig. S12 *A* and *B*).

### Experimental Manipulation of Syntaxin-4 Levels Alters Insulin Release.

We hypothesized that experimentally manipulating syntaxin-4 levels could alter the rate of exocytosis. We have used INS-1E cells that exhibit β-cell-like features, particularly stimulated insulin release. First, we exposed INS-1E cells to H_2_O_2_ to induce oxidative stress, a common feature of diabetogenic conditions. INS-1E cells survived for 12 h postexposure (*SI Appendix*, Fig. S13*A*), at which point the expression of a molecular marker of endoplasmic reticulum stress, such as *Xbp1s* (which induces the unfolded protein response pathway upon oxidative stress; *SI Appendix*, Fig. S13 *B* and *C*), were significantly increased. Concomitantly, H_2_O_2_ (200 μM) upregulated both *Scgn* and *Snap25* mRNA expression. In contrast, *Syt4* mRNA levels were significantly reduced (Fig. 6*H*). Unexpectedly, insulin secretion nearly doubled (Fig. 6*I*).

Next, we have overexpressed syntaxin-4 in INS-1E cells (*SI Appendix*, Fig. S13*D*). Increased syntaxin-4 levels (*SI Appendix*, Fig. S13*E*) led to reduced basal release vs. mock-transfected cells (*P* = 0.017; Fig. 6*J*). Likewise, KCl (90 mM)-induced excitation of INS-1E cells resulted in lower insulin levels extracellularly (*P* = 0.033) relative to mock-transfected controls (note that KCl significantly increased insulin release in the respective groups relative to their baseline). F-actin “stress” fibers were concomitantly formed in KCl-exposed INS-1E cells (*SI Appendix*, Fig. S13*F*). Finally, we used phorbol-ester (PDBu), which triggered quasi-equivalent maximal insulin release irrespective of syntaxin-4 levels (*P* = 0.712; Fig. 6*J*). These data suggest that syntaxin-4 expression inversely affects insulin release in the presence of stable secretagogin and SNAP-25 levels in INS-1E cells.

## Discussion

Here, we identify syntaxin-4 as a binding partner for secretagogin in both mouse and human. Although the binding of both SNAP-25 and syntaxin-4 at the same site could at first be unexpected, secretagogin is not unique in having dual specificity. Several EF-hand-based interaction modules have more than one binding partner (8, 9, 33): Calmodulin forms a complex with Munc13-1 to prime vesicles (34), as well as with Na^+^ (35) and TRP-type Ca^2+^ channels (36), wherein helical peptide ligands bind within a groove of a single domain (*SI Appendix*, Fig. S14*A*). In these complexes, the peptide ligands use a binding mode analogous to the one described here. We have also defined the precise interaction motifs and provide crystal structures of Ca^2+^-laden secretagogin in complex with the target peptide(s). By performing biochemical analyses, we revealed an ~1 order K_d_ difference in secretagogin’s affinity for SNAP-25 vs. syntaxin-4 in the presence of Ca^2+^. The difference in K_d_ suggests that secretagogin can switch from syntaxin-4 to SNAP-25 if its alternative targets are sufficiently close to do so. This event can, we hypothesize, occur at release sites in endocrine tissues or presynapses in neurons, which contain high amounts of SNARE proteins as well as vesicles. Upon repetitive or sustained stimuli, vesicle recruitment from the reserve pool is needed. SNAP-25 could then compete out secretagogin-bound syntaxin-4 given its higher affinity binding, allowing the stationing of vesicles at release sites. Subsequently, release can occur when SNAP-25 decouples from secretagogin. Thus, the coexistence of, the 1:1 stoichiometry, and sequential increase in affinity (K_d_) between secretagogin, syntaxin-4, and SNAP-25 account for a unidirectional chain of molecular movements for the rapid mobilization of a large number of vesicles (37, 38). This notion is also compatible with secretagogin ablation in β-cells primarily impacting 2nd phase insulin release (17, 18, 20).

Secretagogin does not bind syntaxin-1A. This finding is harmonious to the above hypothesis because syntaxin-1A contributes to the immediate release of readily available vesicles (29) in, e.g., neurons. In endocrine cells, however, which operate with compound exocytosis when intracellular Ca^2+^ levels are persistently elevated (38), the molecular composition of the SNARE apparatus is built around VAMP8, SNAP-25, syntaxin-3, and syntaxin-4 (28, 29). Thus, secretagogin binding to syntaxin-4 but not syntaxin-1A is attuned with its putative role in the Ca^2+^ -sensitivity of newcomer vesicle movements during sustained hormone secretion (28, 39–42).

Secretagogin is a modular protein, with the functional significance of its N-terminus remaining unexplored. Considering the presence of many cytoskeletal proteins in secretagogin’s interactome (10, 16, 18), we suggest that this domain could participate in Ca^2+^-independent interactions with the cytoskeleton to act as an anchor for secretagogin at precise locations along the plasma membrane. This hypothesis is consistent with ultrastructural observations in neuroendocrine terminals at the brain/circulation interface (termed median eminence of the hypothalamus), wherein the bulk of secretagogin molecules lines the internal surface of the plasmalemma (14).

Last, the target site for secretagogin in SNAP-25 is inaccessible when SNAP-25 partakes in the four-helix bundle SNARE complex (*SI Appendix*, Fig. S14 *B* and *C*) (3, 43). In syntaxin-4, the target site is the α-helical segment (H_b_), which is not involved in the syntaxin-1A/Munc18 interaction (*SI Appendix*, Fig. S14*D*). Indeed, we show that secretagogin can neither bind to nor dissolve preformed four-helix SNARE complexes. Considering the Ca^2+^-dependent and thus transient nature of the pairwise secretagogin-SNAP-25 interaction, we suggest that secretagogin detachment from membrane-anchored SNAP-25 is a prerequisite for the assembly of the final SNARE complex once a vesicle is present and, consequently, for a release event to take place. Thus, our observations also bear significance for pharmacologically modulating vesicle release by characterizing secretagogin as a chaperon whose targeting could complement SNAP-25 inhibition (e.g., by αSNAP) (2, 44, 45) or by the cysteine string protein α-small glutamine-rich protein-heat-shock cognate 70 complex during vesicle docking (46).

From a technical perspective, we faced a challenge when aiming at the X-ray structures of secretagogin bound to its target peptides. We presented the interacting peptide segments using GFP as a crystallization chaperon. This was necessary because the N-terminal domain A of mammalian secretagogin lacks Ca^2+^-binding (Fig. 3) (15, 16). Consequently, it is flexible and partially disordered. This flexibility impedes the formation of well-diffracting protein crystals, which may be compensated for by the presence of the GFP-tag. Unlike the mammalian secretagogin, zebrafish secretagogin contains intact Ca^2+^-binding sites in each EF-hand motif and could be crystallized without the use of crystallization chaperons (15, 24). In mammalian secretagogin, domains B and C undergo conformation changes upon Ca^2+^ binding, and form a surface groove in domain C for the docking of both SNAP-25 and syntaxin-4-derived peptide ligands. These findings were confirmed by the CavityPlus algorithm (47), which identified this very groove, the most conserved region mapped in 3D (Fig. 6*B*). Binding modes of the peptide ligands are similar, wherein the amphipathic helices fill the binding cavity of secretagogin forming a hydrophobic interface aligned with the same helical phase. One striking feature common to human (*SI Appendix*, Fig. S4), mouse (Fig. 3), and zebrafish secretagogin (24) is the abrupt binding groove when in complex with a SNAP-25-derived peptide. This groove is not compatible with the docking of an elongated helical segment, as the loop bordering the binding groove (residues 189 to 199) blocks access (*SI Appendix*, Fig. S14*E*). Moreover, the H-bonding of G155 is involved in the stabilization of the continuous SNAP-25 helix beyond this position, a contact point occupied by a H-bond acceptor Met190 in secretagogin. Hence, the C-terminal helix of SNAP25 is expected to be bent or partly unfolded upon binding to secretagogin, which likely prevents its involvement in the SNARE complex.

Cumulatively, our study provides structural information on the binding partners and dynamic interactions of secretagogin, and suggests a mechanism for this Ca^2+^-sensor to differentiate the rate and kinetics of hormone release from endocrine cells.

## Materials and Methods

### Design, Cloning and Purification of Recombinant Protein Constructs.

Coding sequences for mouse secretagogin (Uniprot ID:Q91WD9), human secretagogin (Uniprot ID:O76038), mouse SNAP-25-1 and -2 (P60879-1, P60879-2), mouse syntaxin-4 (P70452), human syntaxin-4 (Q12846), mouse syntaxin-1A (O35526), mouse VAMP2 (P63044) and mouse VAMP8 (O70404) were obtained from GenScript, and cloned into a pNIC28Bsa4 expression vector (Genbank ID: EF198106) using upstream *NcoI* and downstream *HindIII* restriction sites. We refer to *SI Appendix* for technical details on the expression and purification of recombinant proteins and the co-expression of protein complexes.

### Pull-down Experiments.

To test mouse secretagogin binding to His-tagged partners, relevant constructs were expressed in *E. coli* BL21(DE3). Cells were lysed, and the His_6_-tagged proteins captured on Ni-NTA resin (Thermo Scientific). His_6_-tagged targets bound to secretagogin constructs were analyzed by SDS-PAGE. As described in *SI Appendix*.

### Protein Crystallization and Structure Determination.

Crystals were produced by the vapor diffusion method in sitting drop format. X-ray diffraction datasets were collected at either 100 K at the BIOMAX beamline of the MAX-IV synchrotron (Lund) or at the European Synchrotron Radiation Facility beamline ID30B. Experimental parameters, data collection, resolution, analysis, and statistics are available in *SI Appendix*. Crystallographic data were deposited at the Protein Data Bank (accession codes 8BAN, 8BAV, 8BBJ).

### Immunohistochemistry.

Mice were deeply anesthetized by isoflurane (see *SI Appendix* for details), transcardially perfused, and pancreata dissected for immunohistochemistry. Superresolution images were captured by an AiryScan detector module (Zeiss). Further details are provided in *SI Appendix*.

### Cell Culture.

Rat insulinoma β-like cells (INS-1E) were transfected with pcDNA-3.1 plasmid (1 µg) using a Nucleofector system (Lonza). Insulin secretion was monitored by ELISA. We refer to *SI Appendix* for additional details.

## Supplementary Material

Appendix 01 (PDF)

Dataset S01 (XLSX)

## Data Availability

Crystallography data were deposited at the Protein Data Bank (PDB) under accession codes 8BAN (48), 8BAV (49), and 8BBJ (50).
