# Supplementary material for: A hydrophobic groove in secretagogin allows for alternate interactions with SNAP-25 and syntaxin-4 in endocrine tissues
Source: Proc Natl Acad Sci U S A. 2024 Apr 9;121(16):e2309211121. doi: 10.1073/pnas.2309211121 (PMC11032447; doi:10.1073/pnas.2309211121)
Supplement: Supplementary file 1 — Appendix 01 (PDF) [file pnas.2309211121.sapp.pdf]

Supporting Information to the manuscript:

A hydrophobic groove in secretagogin allows for alternate interactions with SNAP-25 and syntaxin-4 in endocrine tissues

Edit Szodorai, Zsofia Hevesi, Ludwig Wagner, Tomas Hökfelt, Tibor Harkany and Robert Schnell

This file contains:

Dataset S1. Protein expression constructs used in this study (also available online)
Table S1. Parameters and results of ITC experiments
Table S2. X-ray diffraction data and refinement statistics
Figure S1. The SNARE complex and the molecular structure of its constituents relevant to the present study
Figure S2. Screening for pair-wise interactions between secretagogin and individual domains of SNARE proteins
Figure S3. Identification of target sites for secretagogin in both SNAP-25 and in syntaxin-4
Figure S4. Human secretagogin in complex with a SNAP25-derived peptide
Figure S5. Structural details of the secretagogin-peptide complexes
Figure S6. Mapping syntaxin-4 binding on isolated domains of secretagogin
Figure S7. Interaction between human secretagogin and the H_{abc} domain of human syntaxin-4
Figure S8. Peptide ligand binding by mutant secretagogin variants
Figure S9. Interaction of mouse secretagogin with the complete cytosolic segment of mouse syntaxin-4 made up by the H_{abc} and the SNARE domains
Figure S10. Mass photometry of mouse secretagogin bound to syntaxin-4 monomers made up by the H_{abc} and SNARE domains
Figure S11. Antibodies used for localization of secretagogin interaction partners in pancreatic islets
Figure S12. Secretagogin, SNAP-25, and syntaxin-4 co-localize at subplasmalemmal positions in pancreatic islets
Figure S13. Endoplasmic reticulum stress and morphological responses of INS-1E cells to H_2O_2
Figure S14. Comparison of secretagogin-peptide complexes and structurally-related protein structures

Detailed description of materials and methods

Dataset S1. Protein expression constructs used in this study

Construct	Protein name		Uniprot	Gene name	CDS	Start-Stop	Notes
mSCGN	SEGN_MOUSE	Secretagogin	Q91WD9	Scgn	NM_145399.1	M1-P276	Full-length, in crystal structure with GFPmSNAP25C-peptide
mSCGN-AB	SEGN_MOUSE	Secretagogin	Q91WD9	Scgn	NM_145399.1	M1-E182	N-terminal and middle domains
mSCGN-B	SEGN_MOUSE	Secretagogin	Q91WD9	Scgn	NM_145399.1	M-S89A-E182	Middle domain (S89A mutant)
mSCGN-BC	SEGN_MOUSE	Secretagogin	Q91WD9	Scgn	NM_145399.1	M-S89A-P276	Middle and C-terminal domains, in crystal structure with GFPmSTX4Hb-peptide (S89A mutant)
mSCGN-C	SEGN_MOUSE	Secretagogin	Q91WD9	Scgn	NM_145399.1	M-E182-P276	C-terminal domain
mSCGN-M7	SEGN_MOUSE	Secretagogin	Q91WD9	Scgn	NM_145399.1	M1-P276	Full-length, R198A mutant
mSCGN-M8	SEGN_MOUSE	Secretagogin	Q91WD9	Scgn	NM_145399.1	M1-P276	Full-length, M229A mutant
mSCGN-M9	SEGN_MOUSE	Secretagogin	Q91WD9	Scgn	NM_145399.1	M1-P276	Full-length, M229E mutant
mSCGN-M10	SEGN_MOUSE	Secretagogin	Q91WD9	Scgn	NM_145399.1	M1-P276	Full-length, V233A mutant
mSCGN-M11	SEGN_MOUSE	Secretagogin	Q91WD9	Scgn	NM_145399.1	M1-P276	Full-length, V233E mutant
mSCGN-M12	SEGN_MOUSE	Secretagogin	Q91WD9	Scgn	NM_145399.1	M1-P276	Full-length, L270A mutant

Construct	Protein name		Uniprot	Gene name	CDS	Start-Stop	Notes
hSCGN	SEGN_HUMAN	Secretagogin	O76038	SCGN	NM_006998.3	M1-P276	Full-length, in crystal structure with GFPhSNAP25C-peptide

Construct	Protein name		Uniprot	Gene name	CDS	Start-Stop	Notes
mSN251	SNP25_MOUSE	Synaptosomal-associated protein 25	P60879-1	Snap25	NM_001355254.1	M1-G206	Full-length
mSN252	SNP25_MOUSE	Isoform 2 of Synaptosomal-associated protein 25	P60879-2	Snap25	NM_001291056.1	M1-G206	Full-length
mSN251-1	SNP25_MOUSE	Synaptosomal-associated protein 25	P60879-1	Snap25	NM_001355254.1	M1-G82	N-term helix
mSN251-2	SNP25_MOUSE	Synaptosomal-associated protein 25	P60879-1	Snap25	NM_001355254.1	M1-S98	N-term helix + Cys
mSN251-3	SNP25_MOUSE	Synaptosomal-associated protein 25	P60879-1	Snap25	NM_001355254.1	M1-D140	N-term helix + MidDom
mSN251-4	SNP25_MOUSE	Synaptosomal-associated protein 25	P60879-1	Snap25	NM_001355254.1	M-A141-G206	C-term helix
mSN252-1	SNP25_MOUSE	Isoform 2 of Synaptosomal-associated protein 25	P60879-2	Snap25	NM_001291056.1	M1-G82	N-term helix
mSN252-2	SNP25_MOUSE	Isoform 2 of Synaptosomal-associated protein 25	P60879-2	Snap25	NM_001291056.1	M1-S98	N-term helix + Cys
mSN252-3	SNP25_MOUSE	Isoform 2 of Synaptosomal-associated protein 25	P60879-2	Snap25	NM_001291056.1	M1-D140	N-term helix + MidDom
mSN252-4	SNP25_MOUSE	Isoform 2 of Synaptosomal-associated protein 25	P60879-2	Snap25	NM_001291056.1	A141-G206	C-term helix
SN2514-1	SNP25_MOUSE	Synaptosomal-associated protein 25	P60879-1	Snap25	NM_001355254.1	M-A141-N159	Cterm deletion: 1 / Nterm deletion: none
SN2514-2	SNP25_MOUSE	Synaptosomal-associated protein 25	P60879-1	Snap25	NM_001355254.1	M-A141-N169	Cterm deletion: 2 / Nterm deletion: none
SN2514-3	SNP25_MOUSE	Synaptosomal-associated protein 25	P60879-1	Snap25	NM_001355254.1	M-A141-D179	Cterm deletion: 3 / Nterm deletion: none
SN2514-4	SNP25_MOUSE	Synaptosomal-associated protein 25	P60879-1	Snap25	NM_001355254.1	M-A141-N188	Cterm deletion: 4 / Nterm deletion: none
SN2514-5	SNP25_MOUSE	Synaptosomal-associated protein 25	P60879-1	Snap25	NM_001355254.1	M-A141-A199	Cterm deletion: 5 / Nterm deletion: none
SN2514(1)	SNP25_MOUSE	Synaptosomal-associated protein 25	P60879-1	Snap25	NM_001355254.1	MA-D147-G206	Cterm deletion: none / Nterm deletion: 1
SN2514(2)	SNP25_MOUSE	Synaptosomal-associated protein 25	P60879-1	Snap25	NM_001355254.1	MA-E151-G206	Cterm deletion: none / Nterm deletion: 2
SN2514(3)	SNP25_MOUSE	Synaptosomal-associated protein 25	P60879-1	Snap25	NM_001355254.1	MA-G155-G206	Cterm deletion: none / Nterm deletion: 3
SN2514(4)	SNP25_MOUSE	Synaptosomal-associated protein 25	P60879-1	Snap25	NM_001355254.1	MA-A164-G206	Cterm deletion: none / Nterm deletion: 4
SN2514-31	SNP25_MOUSE	Synaptosomal-associated protein 25	P60879-1	Snap25	NM_001355254.1	MA-D147-D179	Cterm deletion: 3 / Nterm deletion: 1
SN2514-32	SNP25_MOUSE	Synaptosomal-associated protein 25	P60879-1	Snap25	NM_001355254.1	MA-E151-D179	Cterm deletion: 3 / Nterm deletion: 2
SN2514-33	SNP25_MOUSE	Synaptosomal-associated protein 25	P60879-1	Snap25	NM_001355254.1	MA-G155-D179	Cterm deletion: 3 / Nterm deletion: 3
SN2514-34	SNP25_MOUSE	Synaptosomal-associated protein 25	P60879-1	Snap25	NM_001355254.1	MA-A164-D179	Cterm deletion: 3 / Nterm deletion: 4
GFPmSNAP2514-33	SNP25_MOUSE	Synaptosomal-associated protein 25	P60879-1	Snap25	NM_001355254.1	MA-G155-D179	In crystal structure with mSCGN and hSCGN

In total, 68 protein constructs were designed and used throughout the experiments presented. Here, Uniprot accession codes, sequences of the exact residue-segments, and peptide-constructs were provided. This list is also available as an editable stand-alone Excel worksheet on-line.

Table S1. Parameters and results of ITC experiments

Cell content	Cell concentration (µM)	Syringe content	Syringe concent-ration (µM)	K_d (M)	s.e.m.
**mSCGN-SNAP-25 interaction**					
mSCGN	10	mSNAP252	80	3,23e-08	4,94e-09
mSCGN	10	mSNAP25C	80	7,63e-09	4,77e-10
mSCGN	25	mSNAP2514-33p	80	4,66e-08	2,64e-09
mSCGN-BC	10	mSNAP25C	80	2,55e-08	4,73e-09
mSCGN-C	10	mSNAP25C	80	3,80e-07	4,18e-08
**mSCGN-syntaxin-4 interaction**					
mSCGN	100	(His6)mSTX43 Habc	1500	1,41e-05	2,17e-06
mSCGN	100	(His6)mSTX431 Ha	800	6,67e-06	6,23e-07
mSCGN	50	(His6)mSTX432 Hb	500	2,09e-07	3,38e-08
mSCGN	50	GFPmSTX43210 Hb10	500	8,26e-07	3,31e-08
mSCGN	50	GFPmSTX4327 Hb7	500	2,75e-07	1,59e-08
mSCGN	50	GFPmSTX4329 Hb9	500	5,41e-07	7,68e-08
**mSCGN mutants**					
mSCGN-(R198A)	10	mSNAP25C	80	7,38e-07	9,67e-08
mSCGN-(M229A)	10	mSNAP25C	80	3,58e-08	6,29e-09
mSCGN-(M229E)	10	mSNAP25C	80	2,01e-07	2,98e-08
mSCGN-(V233A)	10	mSNAP25C	80	2,73e-08	7,84e-09
mSCGN-(L270A)	10	mSNAP25C	80	4,26e-08	7,67e-09
mSCGN-(R198A)	50	(His6)mSTX432 Hb	500	8,08e-07	2,61e-08
mSCGN-(M229A)	50	(His6)mSTX432 Hb	500	1,62e-06	7,51e-08
mSCGN-(M229E)	50	(His6)mSTX432 Hb	500	5,21e-06	7,06e-07
mSCGN-(V233A)	50	(His6)mSTX432 Hb	500	1,25e-07	4,33e-09
mSCGN-(L270A)	50	(His6)mSTX432 Hb	500	1,62e-05	3,33e-06

Data for the ITC analysis of interactions between mouse secretagogin (mSCGN) and either SNAP-25 or syntaxin-4, their protein fragments, and peptide-fusion constructs.

Table S2. X-ray diffraction data and refinement statistics

PDB code	mSCGN-GFP/SNAP-25 peptide 8BAN	hSCGN-GFP/SNAP-25cpeptide 8BAV	mSCGN-GFP/sytaxin-4 peptide 8BBJ
**Data collection***			
Beamline	MAX-IV / BioMAX	MAX-IV / BioMAX	ESRF / ID30B
Space group	$P2_12_12_1$	$P2_12_12_1$	$P2_12_12_1$
**Cell dimensions**			
a, *b*, *c* (Å)	71.66, 121.07, 126.37	70.54, 120.44, 124.07	79.41, 103.10, 121.70
α, β, γ (°)	90.0, 90.0, 90.0	90.0, 90.0, 90.0	90.0, 90.0, 90.0
Resolution (Å)	87.42-2.35 (2.43-2.35)	41.36-2.30 (2.38-2.30)	48.29-2.65 (2.78-2.65)
R_{merge}	0.098 (0.841)	0.103 (0.702)	0.070 (0.678)
$I / \sigma I$	11.9 (2.1)	9.5 (1.9)	8.2 (1.4)
Completeness (%)	99.2 (93.0)	99.8 (99.7)	98.2 (99.5)
Redundancy	7.3 (5.9)	4.5 (4.6)	3.3 (3.4)
**Refinement**			
Resolution (Å)	87.42 – 2.35	41.36-2.30	48.29 – 2.65
No. reflections	43747	45084	28894
R_{work} / R_{free}	0.194 / 0.248	0.188 / 0.261	0.213 / 0.269
No. atoms			
Protein	8013	8174	6909
Ca^{2+} ion	8	8	8
water	369	422	12
B-factors			
protein	48.5	40.7	72.5
ligand/ion	45.9	40.7	72.4
water	44.2	37.0	64.4
R.m.s. deviations			
bond lengths (Å)	0.015	0.012	0.004
bond angles (°)	1.73	1.49	0.76

All structures were solved from diffraction data recorded on a single crystal.
*Values in parentheses are for highest-resolution shell.

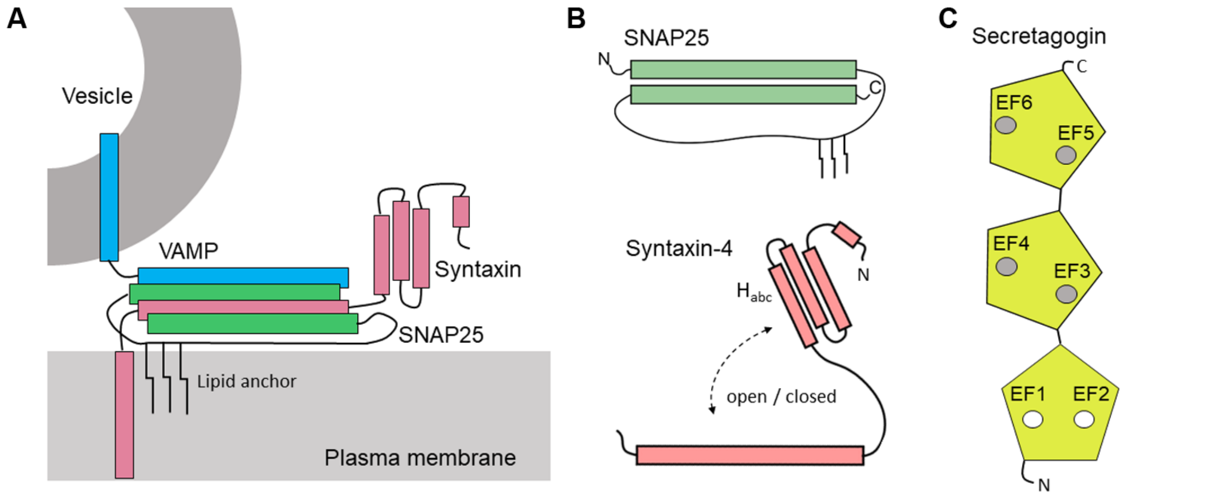

Figure S1. The SNARE complex and the molecular structure of its constituents relevant to the present study

A. Schematic representation of the SNARE complex governing vesicle docking. **B.** Modular organization (domains) of SNAP-25 and syntaxin-4. Dashed line indicates the open and closed conformations of the cytosolic segment of syntaxin-4. **C.** Domain organization of secretagogin. Secretagogin consists of three domains, each containing two EF-hand motifs (EF1-EF6). Ca^{2+} -binding sites in EF1-EF6 were depicted by spheres. Hollow spheres in the EF1 and EF2 motifs indicate that mammalian secretagogin lost Ca^{2+} -binding ability at these sites.

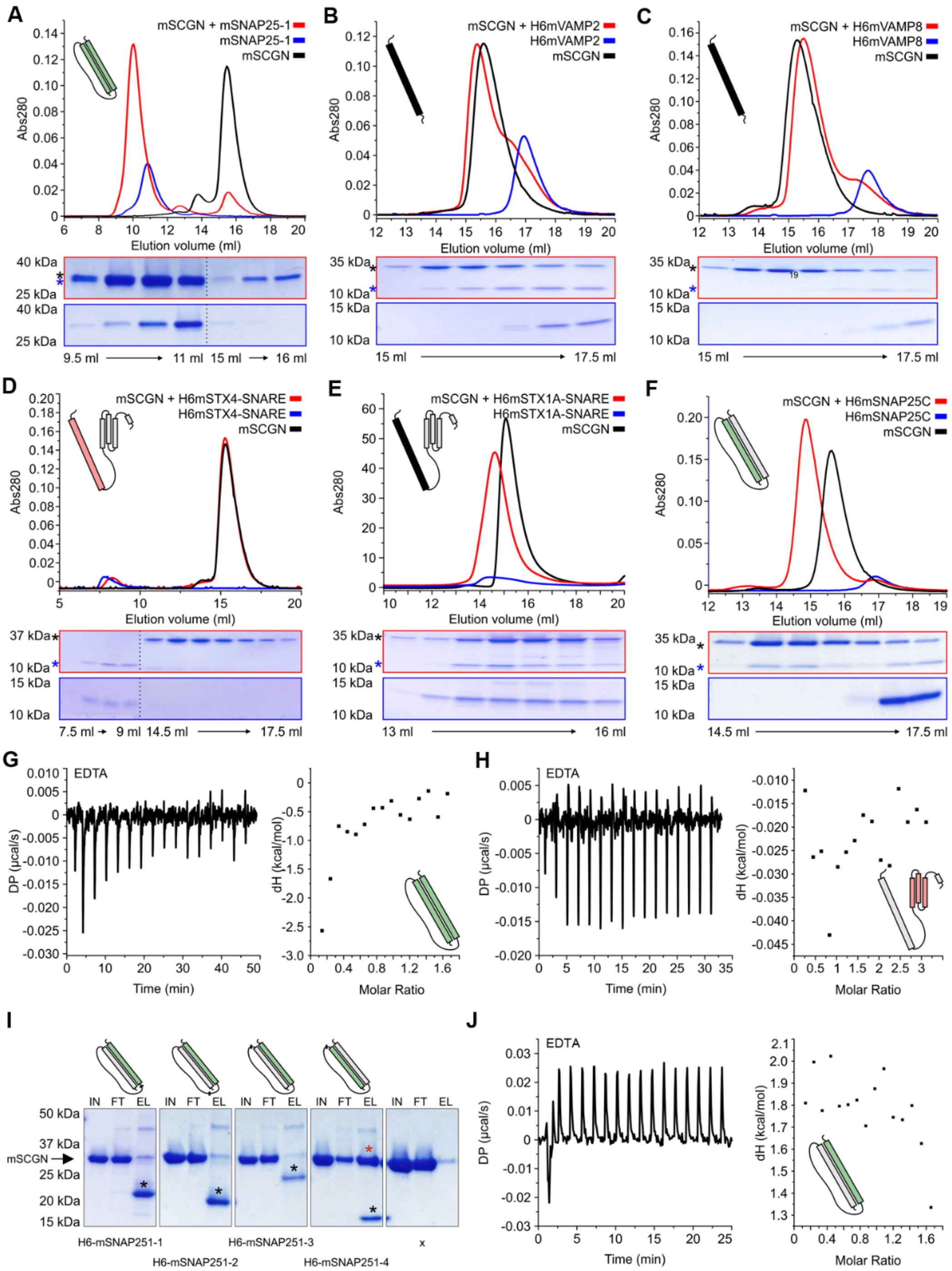

Figure S2. Screening for pair-wise interactions between secretagogin and individual domains of SNARE proteins.

Size exclusion chromatography (SEC) elution profiles to test the potential interaction of mouse secretagogin (mSCGN) with full-length mouse SNAP-25 isoform-1 (**A**), SNARE-helix modules of VAMP2 (**B**), VAMP8 (**C**), syntaxin-4 (**D**), syntaxin-1A (**E**), and the C-terminal helix of the SNAP-25 isoform-1 (**F**). **G,H** ITC recordings under Ca^{2+}-free conditions (with 10 mM EDTA) for mSCGN and either SNAP-25-isoform-2 (**G**) or the H_{abc} domain of syntaxin-4 (**H**). **I**. Pull-down assays identified the fragments of the mouse SNAP-25 isoform-1 (as shown under the gels, with 3D-like sketches above) that interacted with mSCGN. Black asterisks indicate the bands corresponding to a given mSNAP-25 fragment. Red asterisk indicates the band corresponding to mSCGN in a strong interaction with the C-terminal helix of SNAP-25 isoform-1. Note that the sequence of the C-terminal SNAP-25 helix is identical between the two SNAP25 isoforms. **J**. ITC recordings performed under Ca^{2+}-free conditions to test mSCGN binding to the C-terminal helix of SNAP-25.

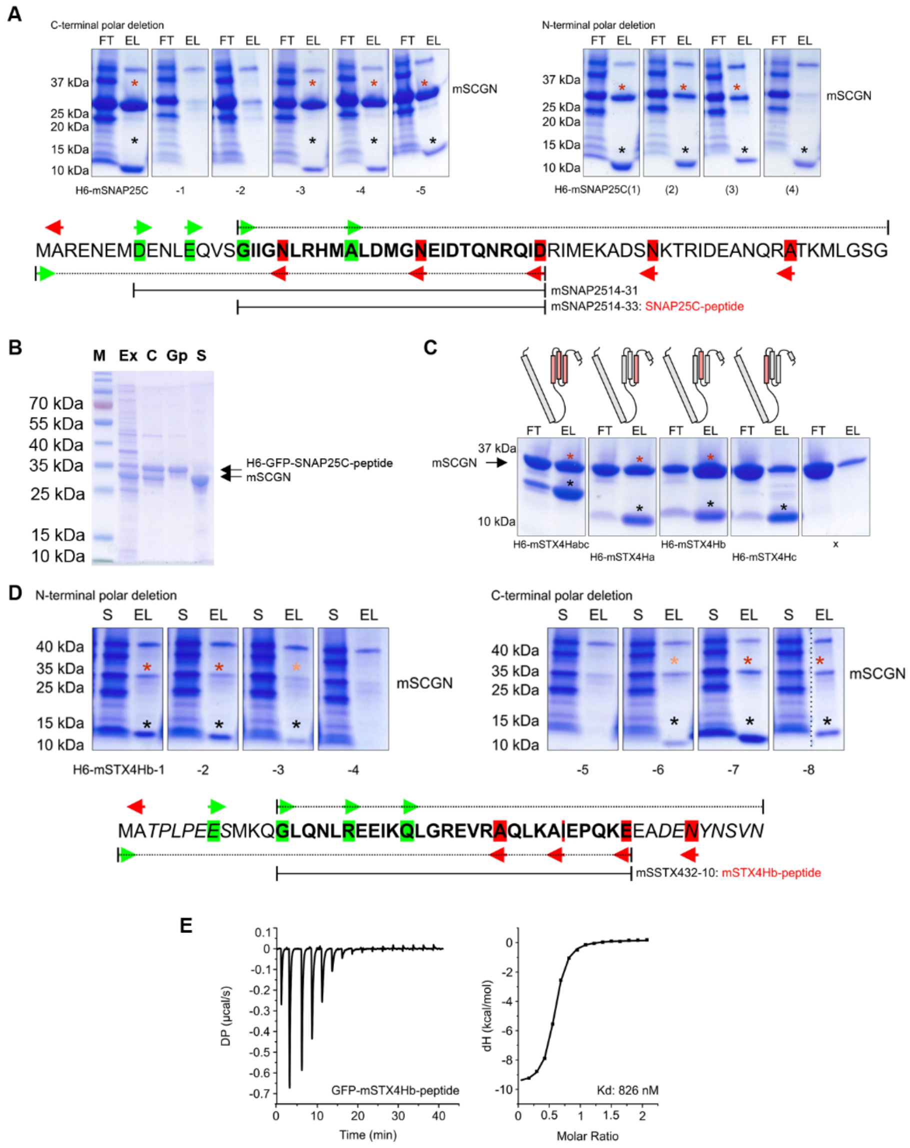

Figure S3. Identification of target sites for secretagogin in both SNAP-25 and in syntaxin-4

A. Pull-down assay to characterize short helical segments as baits that were derived from the C-terminal helix of mouse SNAP-25. Polar deletion constructs were depicted within the amino acid sequence. Highlights in green and red indicate start and stop positions, respectively. Fragments were identified under each SDS gel. Black asterisks denote the protein band corresponding to the particular mouse SNAP-25 fragment. Red asterisks pinpoint the band corresponding to mouse secretagogin (mSCGN) in a strong interaction with a SNAP-25-derived peptide. The shortest N- and C-terminal fragments that retained mSCGN binding, and the two segments that were used in subsequent analysis (SNAP-25$^{14-31}$, SNAP25$^{14-33}$) were shown by dotted and solid lines above and underneath the sequence, respectively. **B.** Co-purification of mSCGN and the His_6-tagged GFP-fusion construct carrying the mouse SNAP-25$^{14-33}$ peptide (sequence in panel A) as C-terminal tag demonstrated that the protein interaction relies on this segment. SDS gels show the total cell protein and purified protein complexes (*C*) made up by the His_6-GFP-SNAP25C-peptide (*Gp*) and the tag-free mSCGN (*S*). **C.** Pull-down assay with His_6-tagged individual helices (H_a, H_b and H_c) derived from the H_{abc} domain of syntaxin-4 (STX4), and used as baits. Black asterisks indicate the protein bands corresponding to the syntaxin-4 fragments. Red asterisks define the band corresponding to mSCGN, and a strong interaction. **D.** Pull-down assay for short His_6-tagged peptides as baits derived from the H_b of the H_{abc} domain of syntaxin-4. Polar deletion constructs were depicted within the amino acid sequence. Green and red colors indicate start and stop positions, respectively. Black asterisks overlain over the SDS gels identify the band corresponding to a given H_b-fragment. Red asterisks indicate the protein band corresponding to mSCGN in a strong interaction with a given peptide. The shortest fragment that retained mSCGN binding and used in later structural studies (mSTX4Hb-peptide) was identified by dotted and solid lines above and underneath the sequence. **E.** ITC recording of the interaction between mSCGN and GFP carrying the mouse syntaxin-4$^{32-10}$-peptide as a C-terminal tag.

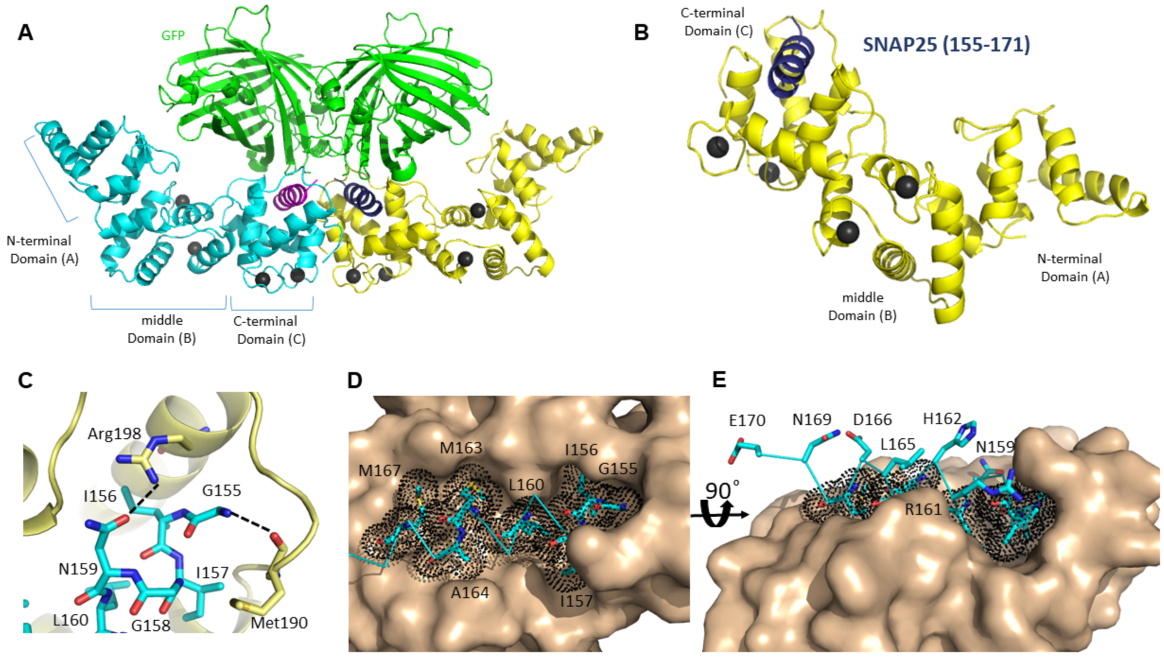

Figure S4. Human secretagogin in complex with a SNAP25-derived peptide

A. Overall structure of human secretagogin (hSCGN) in complex with a GFP-SNAP-25 peptide fusion protein (designated as hSCGN-GFP-SN25^{1-4-33}). The two copies of the complex represent the asymmetric unit (ASU) of the $P2_12_12_1$ crystal form. GFP chains were depicted in green, fused SNAP-25-derived peptides are in dark blue and pink, while hSCGN chains are in cyan and yellow. Gray spheres represent Ca^{2+} ions. **B.** A cartoon of hSCGN (yellow) with the helical SNAP-25-derived peptide (dark blue) bound at the groove of the C-terminal domain. Ca^{2+} ions present in the B and C domains of hSCGN were shown as gray spheres. **C.** Hydrogen bonds formed between hSCGN (beige carbons), and the SNAP-25-derived peptide (cyan carbons) were indicated by dashed lines. **D.** The hydrophobic contact area of the SNAP-25-derived peptide, wherein residues I156, I157, L160, M163, A164, M167 (cyan carbons) were depicted as sticks, filled the binding groove (surface). The dot-surface represents Van der Waals radii. **E.** Side view of the peptide ligand (cyan carbons) at the binding groove of hSCGN (surface) with polar sidechains of the bound SNAP-25-derived peptide (N159, R161, H162, D166, N169, E170) located at the surface.

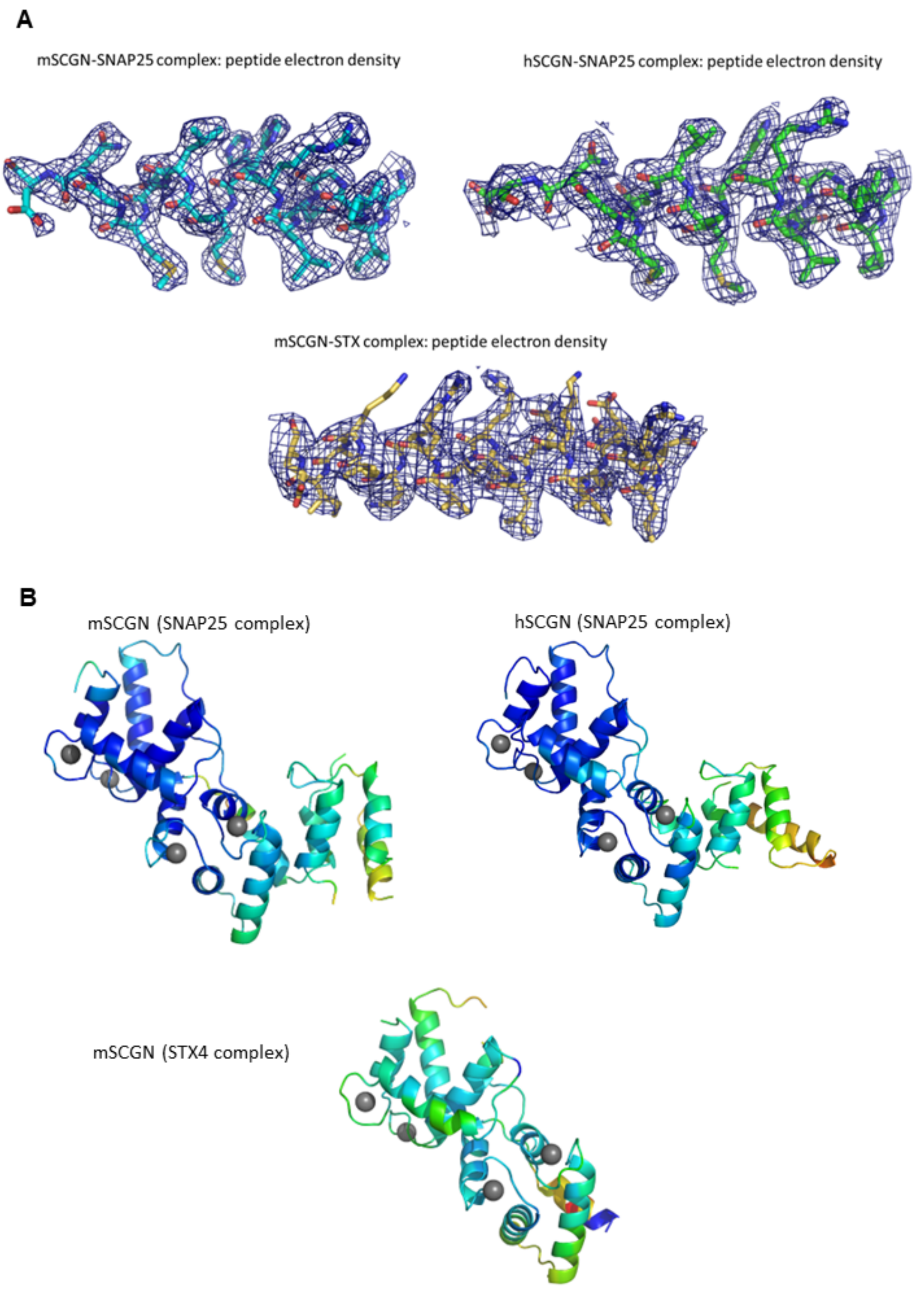

Figure S5. Structural details of the secretagogin-peptide complexes

A. $2F_o$-F_c electron density map at the area of the bound peptide ligands shown as a blue mesh contoured at 1σ for the three peptide complexes of mouse (mSCGN) and human secretagogin (hSCGN), as indicated. **B.** Secretagogin structures colored in the blue-red spectrum according to crystallographic B-factors indicate structural rigidity (dark blue) *vs*. high flexibility (red).

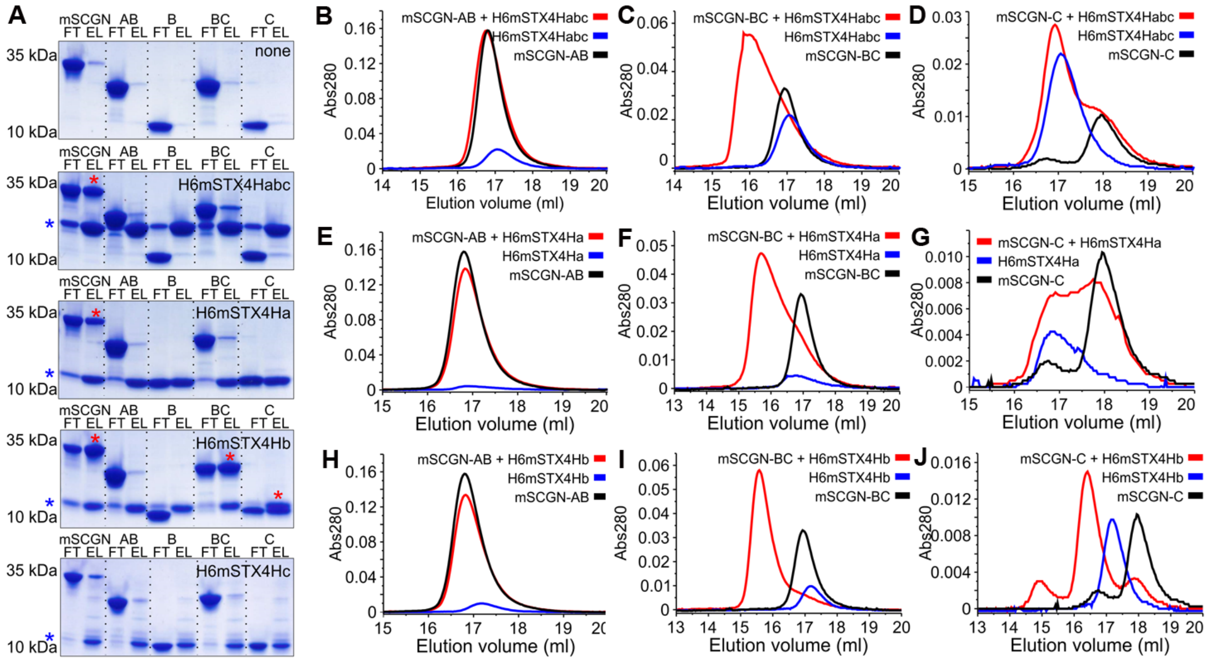

K

	mSCGN		mSCGN-AB		mSCGN-B	mSCGN-BC		mSCGN-C	
	PD	SEC	PD	SEC	PD	PD	SEC	PD	SEC
H6mSTX4Habc	+	+	-	-	-	n.c.	+	-	-
H6mSTX4Ha	+	+	-	-	-	n.c.	+	-	-
H6mSTX4Hb	+	+	-	-	-	+	+	+	+
H6mSTX4Hc	-	-	-	n.a.	-	-	n.a.	-	n.a.

Figure S6. Mapping syntaxin-4 binding on isolated domains of secretagogin

A. Pull-down assay for interactions between His-tagged syntaxin-4 constructs and domain-based constructs of tag-free mouse secretagogin (mSCGN). Full-length mSCGN and constructs harboring its AB, B, BC, and C domains were analyzed as shown on each panel of SDS gels. His$_6$-tagged fragments of syntaxin-4 were used as bait, and consisted of either the entire H_{abc}-domain or smaller constructs corresponding to individual helices (H_a, H_b, H_c). Red asterisks indicate bands corresponding to mSCGN with strong interactions between full-length mSCGN and either the H_a or H_b helices. The H_b helix retained binding to the BC- and C-domain constructs of mSCGN. **B-J**. Elution profiles after size exclusion chromatography (SEC) of paired protein combinations with the constructs tested in pull-down assays. **K.** Checkerboard summary of the results from both pull-down assays and SEC-based separation. Pink fields and '+' signs identify interactions between pairs of protein fragments. Columns and rows correspond to mSCGN domains and syntaxin-4-derived fragments, respectively. In summary, the H_b helix of syntaxin-4 was found to interact with both the C- and BC-domain constructs of mSCGN. *Abbreviations*: H6mSTX4H(x), His$_6$-tagged mouse syntaxin-4 H(x) helix; mSCGN-(x), mouse secretagogin fragments; n.a., not analyzed; n.c., inconclusive; PD, pull-down.

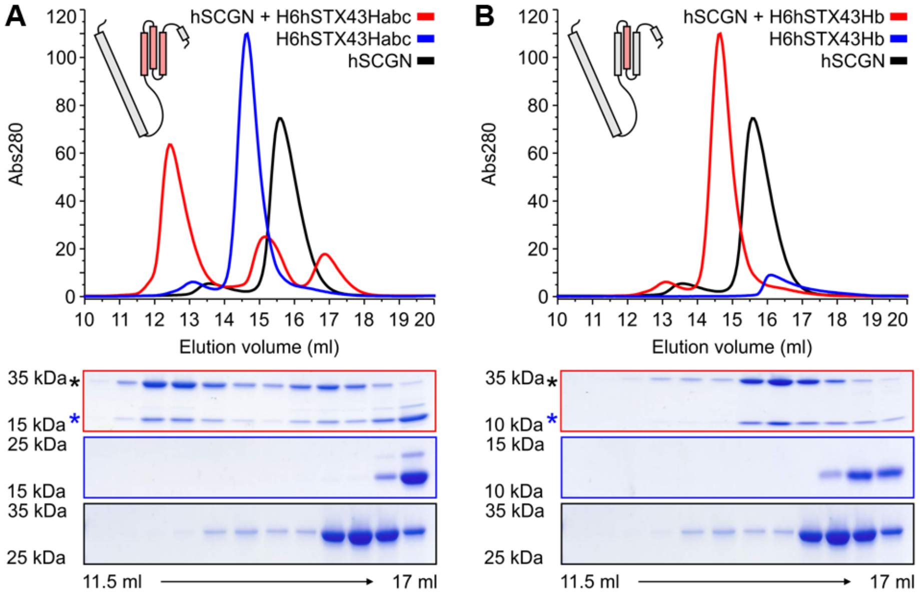

Figure S7. Interaction between human secretagogin and the H$_{abc}$ domain of human syntaxin-4

Top: Size exclusion chromatography (SEC) elution profiles of the interactions between human secretagogin (hSCGN) and either the H$_{abc}$ domain of human syntaxin-4 (H6hSTX43Habc; **A**) or the H$_{b}$-helix alone (H6hSTX43Hb; **B**). *Bottom*: SDS gels with peak areas shown under each SEC plot.

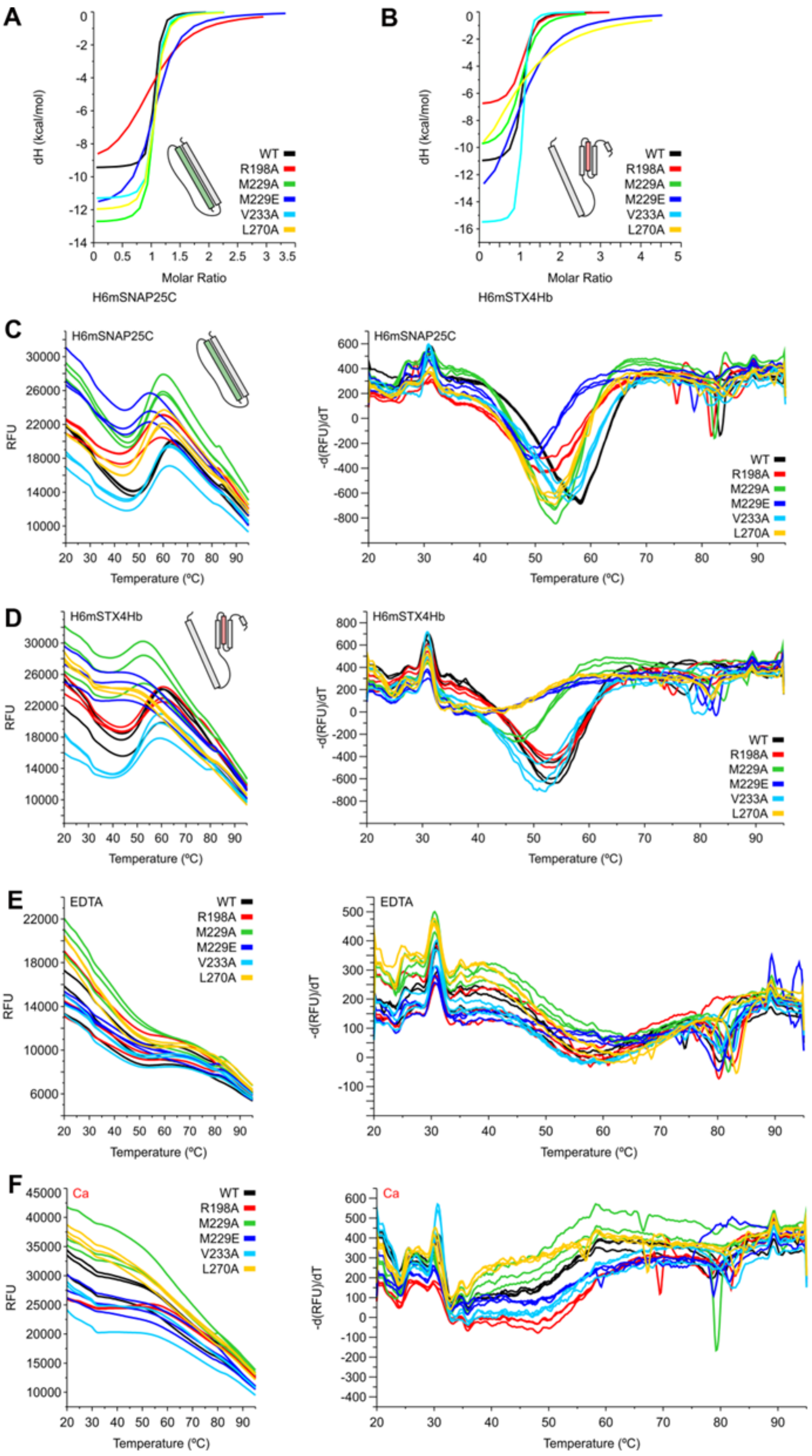

Figure S8. Peptide ligand binding by mutant secretagogin variants

Binding curves in ITC experiments in which the interaction between mutant variants of mouse secretagogin (mSCGN; color-coded for visual clarity) and either the SNAP-25C helix (**A**) or the syntaxin-4 H_b fragment (**B**) were analyzed. **C,D**. Differential scanning fluorimetry showing the denaturation kinetics (*left*) and their derivatives (-dF/dT; *right*) for mSCGN mutants with either mSNAP-25C (**C**) or syntaxin-4 H_b-derived ligand (**D**). Triplicate measurements were plotted. **E,F** Thermal denaturation kinetics of mSCGN mutants in either Ca^{2+}-free- (**E**) or Ca^{2+}-bound (**F**) states. (*left*). The thermal denaturation curves and their derivatives (-dF/dt; *right*) revealed comparable thermal stability of the wild-type protein (black trace) relative to mutants (indicated by the colors), suggesting that no major structural effect can be attributed to the point mutations.

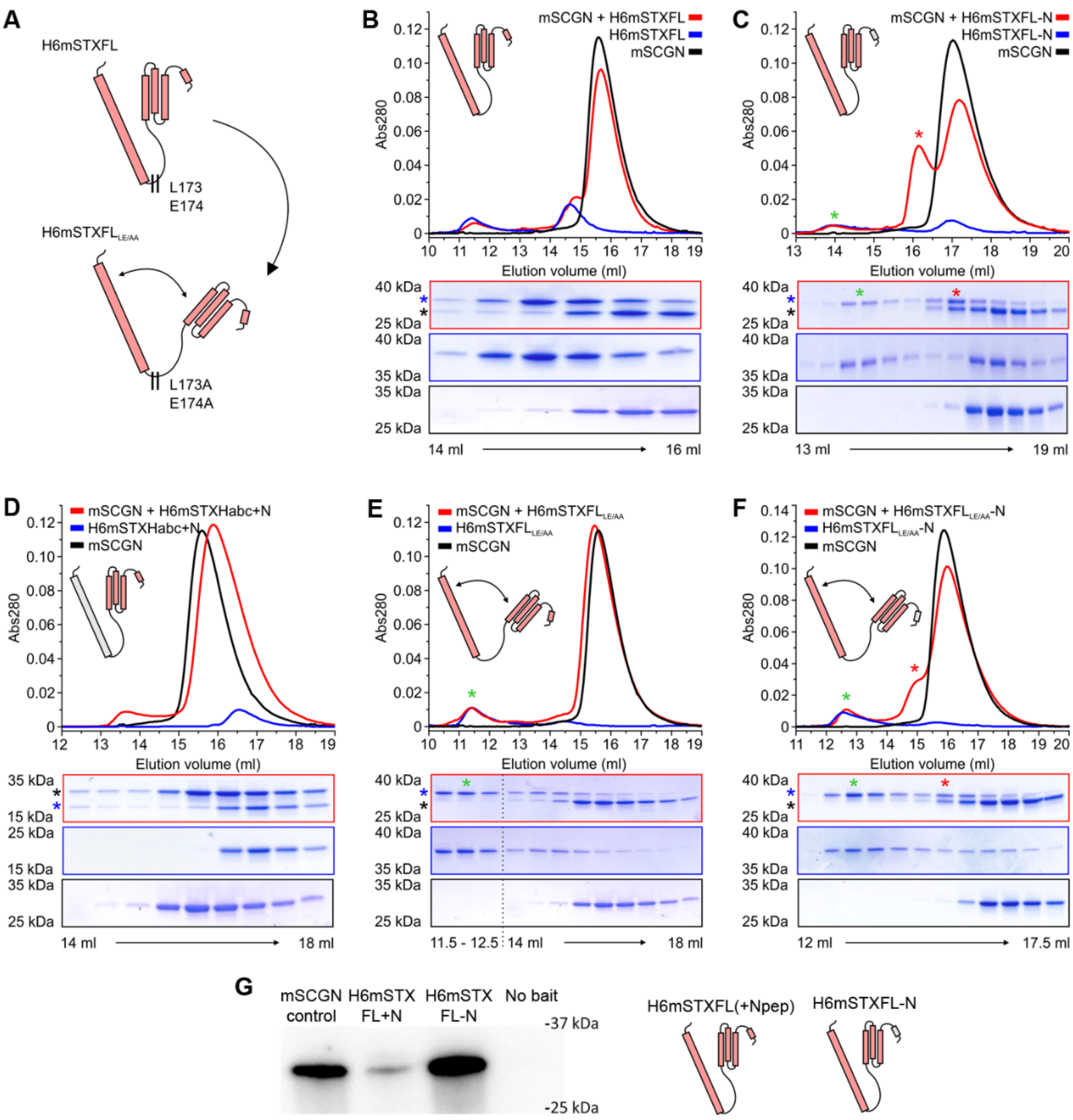

Figure S9. Interaction of mouse secretagogin with the complete cytosolic segment of mouse syntaxin-4 made up by the H_{abc} and the SNARE domains

A. Syntaxin-4 (STX4) exists in both open and closed conformation, depending on the relative positions of its SNARE helix and H_{abc} domain. Mutations in the hinge region between the SNARE and H_{abc} domains (L173A/E174A) restrict mobility, thereby locking the protein in its open conformation. **B-F**. Various constructs of mouse syntaxin-4 (mSTX4) were tested for their binding to mouse secretagogin (mSCGN) using size exclusion chromatography (SEC). Sketches of the protein constructs tested are shown in each panel, wherein pink color identifies the segments included. Missing segments were depicted in gray. Representative SDS gels of the peak areas are under each SEC plot. Red and green asterisks indicate monomeric and oligomeric mSTX4, respectively. **B.** SEC profile of the complete cytosolic segment of mSTX4 in its closed conformation, including the N-peptide, H_{abc} domain, wild-type linker, and SNARE helix. **C.** Same as in (B) but without the N-peptide. **D.** H_{abc} domain with the N-peptide. **E.** The complete cytosolic segment of mSTX4 in its open conformation, including the N-peptide, H_{abc} domain, mutant linker, and SNARE helix. **F.** Same as (E) without the N-peptide. **G**. mSCGN binding to immobilized His-tagged syntaxin-4 constructs harboring the H_{abc}-domain and the SNARE-helix with or without the N-peptide (H6mSTXFL+N vs. H6mSTXFL-N, depicted to the right). Western blotting was used to detect bound mSCGN, and revealed an inhibitory effect of the N-peptide of syntaxin-4.

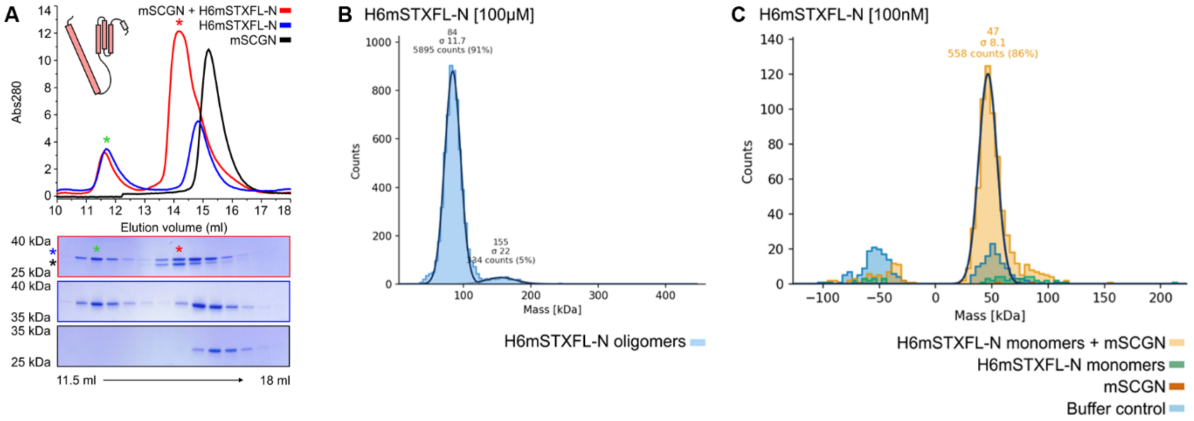

Figure S10. Mass photometry of mouse secretagogin bound to syntaxin-4 monomers made up by the H_{abc} and SNARE domains

A. (*top*) Size exclusion chromatography (SEC) profile showing syntaxin-4 oligomers (asterisk in green), and the preference of mouse secretagogin (mSCGN) for mouse syntaxin-4 monomers (H6mSTXFL-N) as binding partner (asterisk in red). (*bottom*) Representative SDS gels aligned with peak areas in the SEC plot. **B.** Mass photometry data plotted as histograms for the complete cytosolic segment of mouse syntaxin-4 (H6mSTXFL-N) in its closed conformation (wild-type linker sequence), including the SNARE-helix, the H_{abc} domain but lacking the N-peptide. These data confirmed the existence of syntaxin-4 oligomers (84 kDa) at 100 µM protein concentration (as in the SEC experiments). **C.** Mass photometry data plotted as histograms revealed monomers of the cytosolic segment of syntaxin-4 in complex with mSCGN at 100 nM protein concentration, where syntaxin-4 oligomers are unlikely to exist.

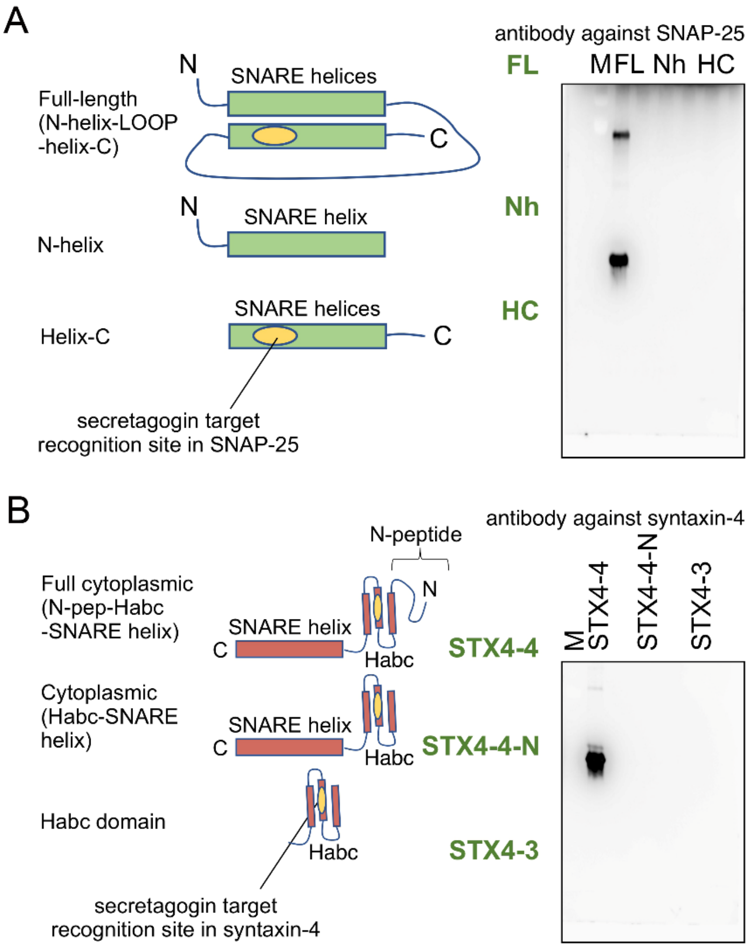

Figure S11. Antibodies used for localization of secretagogin interaction partners in pancreatic islets.

When evaluating protein localization by histochemistry, the position of epitopes can determine if proteins that directly interact can be detected because of the accessibility of antibody binding domains. Therefore, we tested if the putative binding sites between secretagogin and either SNAP-25 or syntaxin-4 could bias histochemical detection. **A.** SNAP-25 was detected by a polyclonal antibody made in rabbit (#111 002, Synaptic Systems) with chromogenic detection (swine anti-rabbit HRP-conjugated secondary antibody, 1:300; Dako, #P0217). Three recombinant constructs of mouse SNAP-25 were used to map the region recognized by this antibody. While full-length SNAP-25 was recognized, neither the N-helix nor the C-helix, the latter containing the secretagogin target segment, was detected. Thus, this antibody recognizes the inter-helix loop of the protein and does not interfere with the detection of secretagogin-SNAP-25 complexes. **B.** Three constructs of mouse syntaxin-4 were used for epitope mapping. Syntaxin-4 was detected by a polyclonal antibody made in rabbit (#AB5330; Merck) with secondary antibody detection and amplification as above. This primary antibody recognized the full cytoplasmic portion of syntaxin-4. However, neither a truncation mutant (with amino acid (AA) residues 1-31 in the N-peptide missing) nor the H_{abc} domain alone, which contains the secretagogin target site, was recognized. These data confirm the supplier's description of the antibody recognizing AA2-23 in the N-peptide segment. Thus, this antibody will not be affected by epitope interference upon complex formation with secretagogin either.

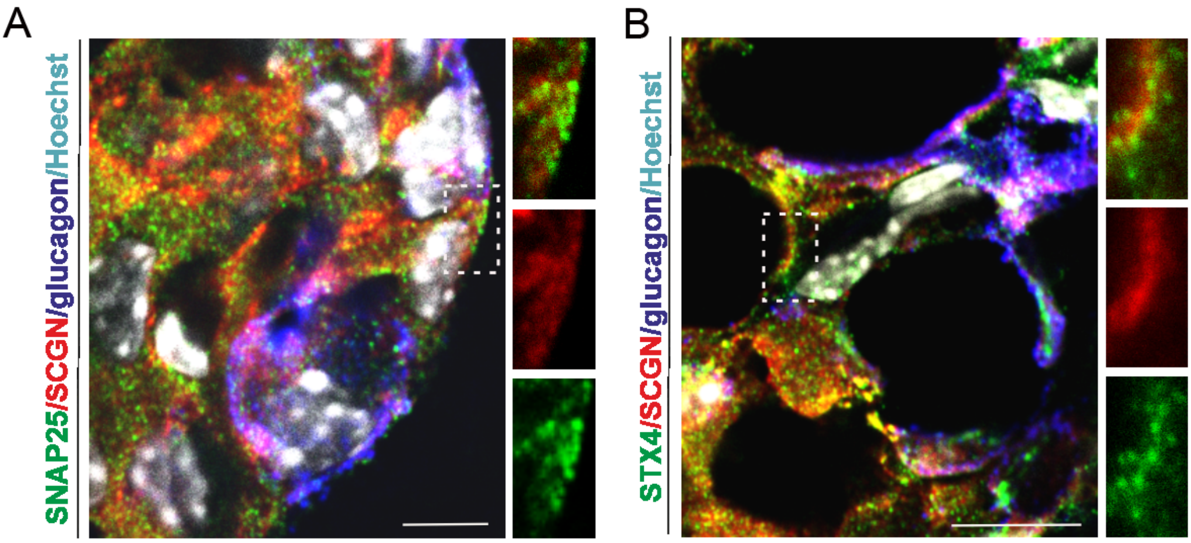

Figure S12. Secretagogin, SNAP-25, and syntaxin-4 co-localize at subplasmalemmal positions in pancreatic islets

A. Co-localization of secretagogin (SCGN) and SNAP-25 in the islands of Langerhans (iL). Both proteins were localized in virtually all cells of pancreatic islets at subplasmalemmal compartments. **B**. Co-localization of SCGN and syntaxin-4 (STX4) Syntaxin-4 was also indiscriminately present in endocrine cells. Open rectangles identify the general location of the insets. Sections were counterstained to visualize glucagon in α-cells and Hoechst 33,342, a nuclear dye. *Scale bars* = 5 μm.

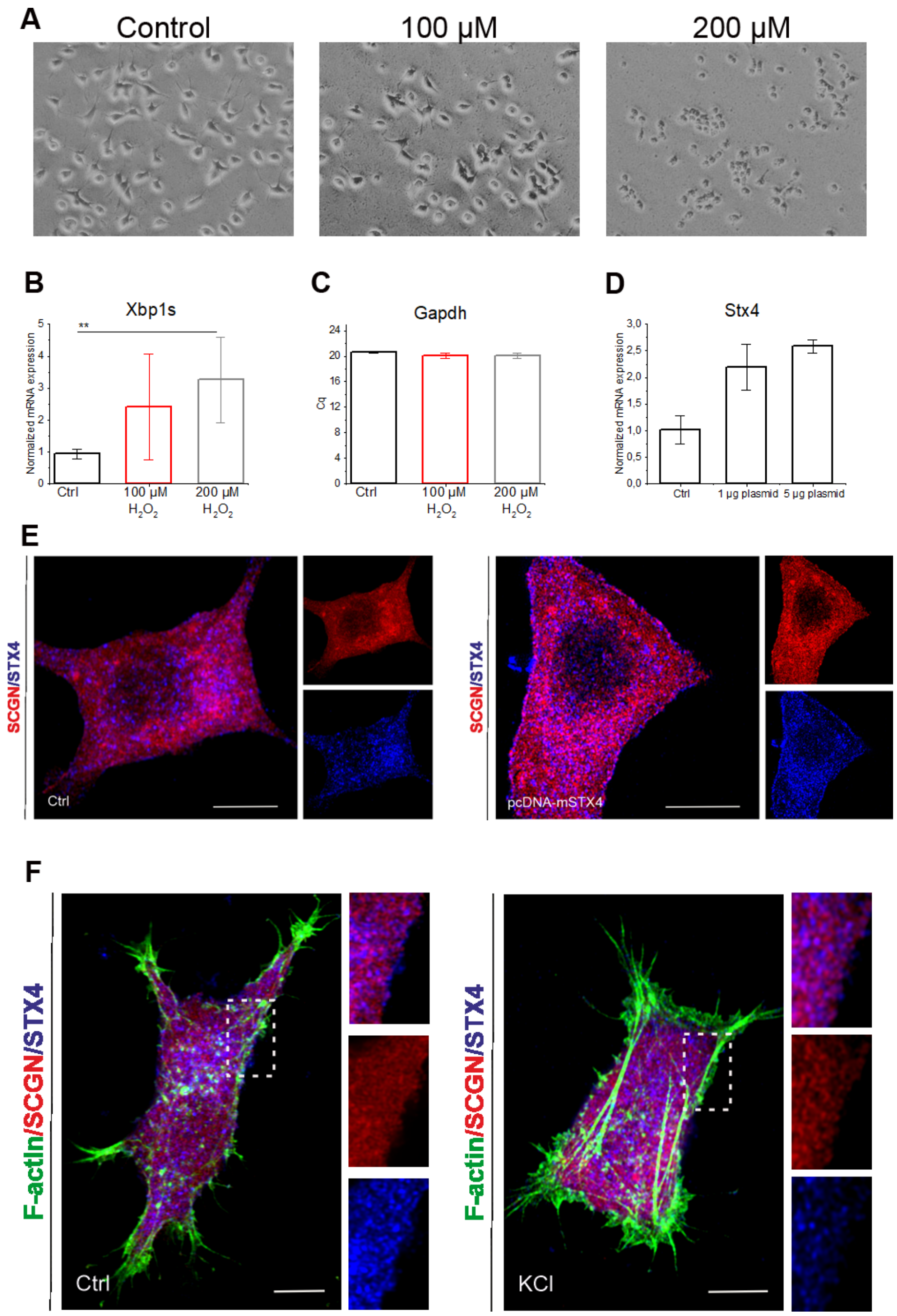

Figure S13. Endoplasmic reticulum stress and morphological responses of INS-1E cells to H_2O_2

A. Photomicrographs of INS-1E cells representative to control (vehicle-treated) and H_2O_2-exposed conditions. H_2O_2 was used at 100 µM and 200 µM concentration for 12 h. Note that 200 µM H_2O_2 led to cellular shrinkage. **B**. mRNA expression of *Xbp1s*, an ER-stress marker, were elevated in a concentration-dependent manner (100 µM *vs.* 200 µM; $^{**}p < 0.01$). **C**. *Gapdh* expression in INS-1E cells (control *vs.* H_2O_2-treated) were unchanged, as shown by the equivalent quantification of cycle (Cq). **D**. The efficacy of syntaxin-4 overexpression (1 µg or 5 µg of pcDNA-STX4 plasmid) was confirmed by qPCR. *Gapdh* was used as internal control. **E**. Cellular localization of secretagogin (SCGN) and syntaxin-4 (STX4) in INS-1E cells that had been transfected either with a mock (control) or overexpression construct. STX4 accumulated particularly in the subplasmalemmal compartment of the transfected cells. **F**. F-actin-labelling in INS-1E cells showed stress-fiber-like cytoskeletal structures upon KCl stimulation. At the same time, syntaxin-4 levels were reduced. *Scale bars* = 5 µm.

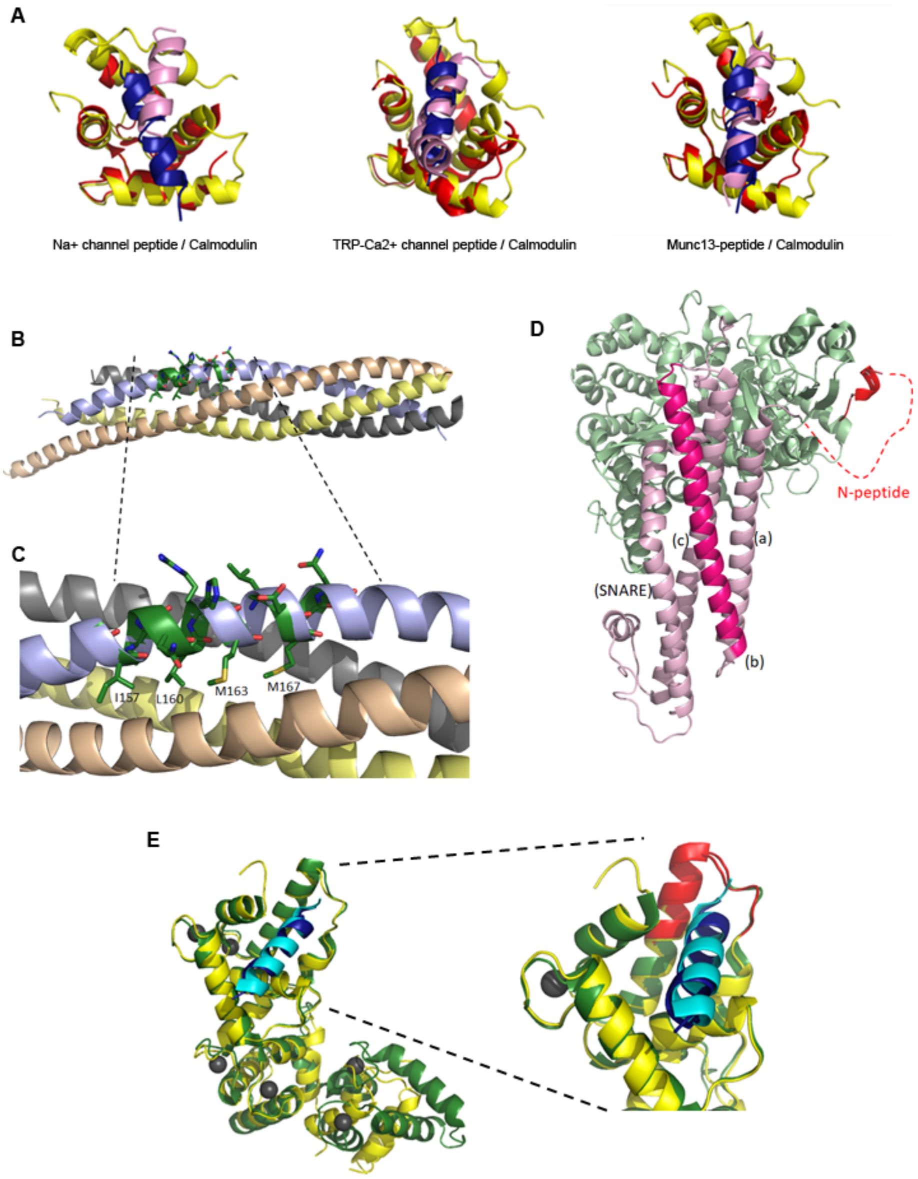

Figure S14. Comparison of secretagogin-peptide complexes and structurally related proteins

A. Comparison of the mouse secretagogin/SNAP-25-derrived peptide complex (in yellow and blue, respectively) with three calmodulin-peptide complexes (in red, pink) reveals similarity in binding mode, with a groove formed in a single domain between two EF-hand motifs. **B,C.** Four-helix bundle of the SNARE complex comprising SNARE helices of syntaxin-1A, VAMP-2 and SNAP25 (PDB: 1N7S). The target site of secretagogin in SNAP-25 is highlighted by displaying the AA residues as green sticks in the close-up in panel (**C**). **D.** Cartoon of the Munc-18 (gray)/syntaxin-1A (pink, magenta) complex (PDB:3C98). The binding mode of syntaxin-1A shows that the N-peptide (in red) is stretched out, mostly disordered except a short fragment situated far away from the remaining fragment of the protein. The Munc-18 interaction involves the SNARE-helix and helices H_a and H_c from the H_{abc} domain. Helix H_b (magenta) where the target site of secretagogin is in syntaxin-4 does not interact directly with Munc-18. **E.** Superposition of mouse secretagogin (in yellow) and the SNAP-25-derived peptide (in blue) with its counterpart from zebrafish (in green and cyan). Close-up on the peptide-binding groove (*right*) with the loop involving secretagogin residues 189-199 (mouse sequence numbering) highlighted in red. This loop can block the docking of a continuous helical peptide.

Methods (detailed description of the experimental methods)

Design and cloning of recombinant protein constructs

Coding sequences for mouse secretagogin (Uniprot ID:Q91WD9), human secretagogin (Uniprot ID:O76038), mouse SNAP-25-1 and -2 (P60879-1, P60879-2), mouse syntaxin-4 (P70452), human syntaxin-4 (Q12846), mouse syntaxin-1A (O35526), mouse VAMP2 (P63044) and mouse VAMP8 (O70404) were obtained from GenScript and cloned into a pNIC28Bsa4 expression vector (Genbank ID: EF198106) using upstream *NcoI* and downstream *HindIII* restriction sites. For co-expression, mouse and human secretagogin coding sequences were subcloned into a pACYC-Duet vector (Merck) using the same restriction sites. Domain-constructs and protein fragments of mouse secretagogin and partner proteins were prepared by amplifying their coding sequences by PCR using Pfu-Turbo polymerase (Agilent), appropriate amplification primers with the full-length clones as templates, and subsequent cloning into pNIC28Bsa4 vector using ligation-independent cloning (1). pNIC28Bsa4-based expression constructs contain an N-terminal hexahistidine (His_6)-tag and a tobacco etch virus (TEV) protease recognition site (MHHHHHHSSGVDLGTENLYFQ*S; * *indicates the cut position*) for affinity tag removal. Recombinant proteins carried one serine residue on their N-terminus after affinity tag removal by TEV protease. pACYC-Duet1-based constructs contained no additional tag-sequences. Full-length mouse syntaxin-4 was expressed in INS-1E cells from a pcDNA3.1 expression vector (Invitrogen), wherein the coding sequence was cloned with upstream *NheI* and downstream *XhoI* sites.

N- and C-terminal polar deletions of mouse SNAP-25 and mouse syntaxin-4 were produced based on their full-length coding sequences as templates using construct-specific primers designed with 3' and 5' phosphate modifications and PCR-amplification using Pfu-Turbo polymerase (Agilent).

Point mutations in mouse secretagogin were produced by applying Quickchange or megaprimer mutagenesis (2). pNIC28Bsa4-based mutant constructs amplified by the Quickchange method were purified from the wild-type plasmid template by incubation at 37 °C for 4 h in the presence of DpnI (New England Biolabs) prior to transformation. Amplified mutant constructs produced by megaprimer mutagenesis were gel-purified (GeneJET Gel Extraction kit, ThermoFisher), cleaved with restriction enzymes to produce *NcoI* and *HindIII* sticky ends, and ligated into a pNIC28Bsa4-vector using Instant-Sticky-end ligation (New England Biolabs).

All constructs were transformed into *E. coli* Stellar chemically competent cells (Takara Bio). Plasmid preps were purified using the GenJET plasmid miniprep kit (ThermoFisher). All DNA sequences were confirmed by sequencing (Eurofins Genomics Germany GmbH). A catalog of the 68 protein constructs based on the above 10 target proteins was provided in Table *S1*, which lists their sequences, accession codes, construct borders, engineered mutations, and their use in this work.

Production and purification of the recombinant proteins

Recombinant proteins were produced in *E. coli* BL21(DE3) in 1.5L LB media supplemented with 30 µg/ml kanamycin for pNIC28Bsa4-based constructs or, additionally, 25 µg/ml chloramphenicol for co-expression when using pACYC-Duet1-based expression constructs. Cultures were grown at 37 °C until the OD_{595} reached 0.5-0.6, cooled to 21 °C, and expression was induced by adding isopropyl-β-D-1-thiogalactopyranoside (to 0.1 mM concentration). Protein production was carried out under induction at 21 °C for 18 h. Cells were harvested by centrifugation (4,000 rpm x 30 min), resuspended in a buffer containing 25 mM Tris-HCl, 300 mM NaCl, 10 mM imidazole (pH 8.0), and lysed by the addition of lysozyme (40 µg/mL), DNase-I (6 µg/mL), $MgCl_2$ (1 mM), while sonicated. The lysate was cleared by centrifugation (18,500 rpm, 25 min), and loaded onto a 1 mL Ni-NTA column (Thermo Scientific). After washing with 20 column volume of 25 mM Tris-HCl, 300 mM NaCl, 10 mM imidazole (pH 8.0),

proteins were eluted by an increasing imidazole gradient (25 to 500 mM). Fractions containing high concentration of the target protein were pooled, imidazole was removed by passing through a PD10 desalting column (GE Healthcare) that retained the proteins in 25 mM Tris and 150 mM NaCl (pH 8.0). For affinity tag removal, protein preparations were treated with TEV protease (5 µg/mg target protein) in the presence of 2 mM DTT at 20 °C for 18 h. His_6-tagged TEV protease and un-cleaved proteins were removed by running the samples through a 1 mL Ni-NTA column collecting the processed, tag-free proteins in the flow through. When proteolytic tag removal was not necessary, the N-terminal His_6 tag was left on the recombinant proteins and exploited in interaction tests using pull-down assays, and when using tag-specific antibodies. Pure protein preparations were concentrated using a Vivaspin device (Sartorius) with 10 kDa or 3 kDa molecular weight cut-off to < 2 mL and loaded on size exclusion chromatography columns (SEC; Superdex-200 or -75, GE Healthcare), equilibrated with 25 mM Tris and 150 mM NaCl (pH 8.0). Peak fractions of the recombinant proteins or protein complexes were pooled and concentrated using a Vivaspin device with 10-kDa or 3-kDa cut-off filters to 10-34 mg/mL. Protein preparations were either used immediately for crystallization and follow-up experiments or aliquoted, flash frozen in liquid N_2, and stored at –80 °C. Protein preparations of mouse and human secretagogin for crystallization were supplemented with DTT (2 mM final concentration) throughout the purification process. Ca^{2+} -dependent protein complexes were isolated by the same protocol using buffers supplemented with 2 mM $CaCl_2$. Recombinant proteins were analyzed by both SDS-PAGE and analytical size exclusion chromatography to confirm their purity and oligomerization states.

Analytical size-exclusion chromatography

Analytical SEC as quality control was done using purified proteins (0.5 mg each) on a Superdex-200 Increase GL column (GE Healthcare) equilibrated with the buffer: 25 mM Tris-HCl, 150 mM NaCl (pH 8.0) at a flow rate of 0.5 ml/min with the absorbance monitored at 280 nm. Peak elution volumes were used to estimate the mass of the oligomers based on calibration using ribonuclease-A (13.7 kDa), chimotrypsinogen-A (25 kDa), ovalbumnin (43 kDa), and albumin (67 kDa). The void volume was determined by blue-dextran (2 MDa).

The analysis of Ca^{2+}-dependent interactions in SEC was carried out as above using a buffer supplemented with 2 mM $CaCl_2$ (25 mM Tris-HCl, 150 mM NaCl, 2 mM $CaCl_2$ at pH 8.0). Proteins were prepared at 100 µM final concentration in the same buffer and incubated at 21 °C for 15 min prior to binding. Fractions were analyzed by SDS-PAGE.

A four-helix bundle SNARE complex was generated to assess secretagogin binding to the C-terminal SNARE domain of SNAP-25 when assembled in a SNARE complex. Purified SNARE-domain constructs of syntaxin-4, VAMP2, and SNAP25 (all from mouse) were mixed at 69 µM each in 200 µL volume, incubated at 4 °C for 18 h, and loaded onto analytical Superdex-200 SEC columns equilibrated with 25 mM Tris-HCl and 150 mM NaCl (pH 8.0). Peak fractions containing the four proteins were concentrated on a NanoSep™ (Pall) protein concentration device with 10 kDa cut-off. A 25 µL fraction of the concentrated sample was mixed with mouse secretagogin (100 µM final concentration) in a buffer supplemented with 2 mM $CaCl_2$ (25 mM Tris-HCl, 150 mM NaCl, 2 mM $CaCl_2$ at pH 8.0). Following incubation at 22-24 ºC for 15 min, the sample was loaded onto pre-equilibrated analytical Superdex-200 SEC columns.

To test the effect of secretagogin binding to the H_{abc} domain of syntaxin-4 on SNARE complex assembly, mouse secretagogin and full length syntaxin-4 in open conformation (L173A/E174A double mutant, Fig. *S9A*) were prepared at 100 µM final concentration (200 µL volume) in a buffer supplemented with 2 mM $CaCl_2$ (25 mM Tris-HCl, 150 mM NaCl, 2 mM $CaCl_2$ at pH 8.0). The sample was incubated at 22-24 ºC for 15 min and loaded onto a pre-equilibrated analytical Superdex-200 SEC column. Peak fractions containing the secretagogin-syntaxin-4 complex were concentrated on a NanoSep™ (Pall) protein concentration device (10 kDa cut-off). The secretagogin-syntaxin-4 complex was mixed with SNARE domains of VAMP2 and SNAP-25 at a final concentration of

about 10 μM in 200 μL buffer supplemented with 2 mM $CaCl_2$ (25 mM Tris-HCl, 150 mM NaCl, 2 mM $CaCl_2$ at pH 8.0), incubated at 22-24 °C for 15 min, and loaded onto a pre-equilibrated analytical Superdex-200 SEC column.

Pull-down experiments

To test mouse secretagogin binding to mouse SNAP-25-1 and -2 domains, His-tagged mouse SNAP-25 constructs were expressed in 5 ml LB cultures as above. Two x 2 ml of the cultures were harvested by centrifugation and freeze-ruptured before lysis. Proteins were extracted using BugBuster lysis buffer (Novagen-Merck) supplemented with 2 μg/ml DNase I and 10 mM imidazole. Cell lysates were cleared by centrifugation at 13,200 rpm at 4 °C for 1 min, and subsequent filtration through 0.2 μm filters. His_6-tagged proteins form 2 ml bacterial culture were captured on Ni-NTA beads (Thermo Scientific) at 4°C by being incubated for 45 min, and after 2 washing steps with 10x bead volume of 10 mM and 5x bead volume of 25 mM imidazole in binding buffer (25 mM Tris-HCl, 300 mM NaCl at pH 8.0). Proteins were eluted with 4x bead volume of 300 mM imidazole in binding buffer. For the washing and elution steps, the samples were centrifuged at 8,000 rpm at 4 °C for 1 min. Protein concentration of the eluted fraction was determined by the Bradford assay, and used as an estimate for the interaction study using proteins extracted from the second 2 ml culture. His_6-tagged proteins from the second 2 ml culture were also captured on Ni-NTA beads and after a washing step using 10x bead volume of 10 mM imidazole in binding buffer, the His-tagged protein-bound beads were reconstituted in 4x bead volume of 10 mM imidazole-containing binding buffer supplemented with 2 mM $CaCl_2$ containing non-tagged mouse secretagogin at a concentration corresponding to those of the estimated His_6-tagged target proteins. Samples were incubated at 21 °C for 15 min to allow protein complex formation. Unbound proteins were collected by centrifugation at 8,000 rpm at 4 °C for 1 min (flow-through). The samples were washed in two steps using 10x bead volume of 10 mM and 5x bead volume of 25 mM imidazole in binding buffer (25 mM Tris-HCl and 300 mM NaCl at pH 8.0). His-tagged targets and non-tagged mouse secretagogin if bound to the target were eluted with 4x bead volume of 300 mM imidazole in binding buffer. For the washing and elution steps, each buffer was supplemented with 2 mM $CaCl_2$. Samples were centrifuged at 8,000 rpm at 4 °C for 1 min. Input samples in which His-tagged protein-bound beads were reconstituted, flow-through, and eluted fractions were analyzed by SDS-PAGE.

For testing if mouse secretagogin binds short mouse SNAP-25 C-terminal and mouse syntaxin-4Hb constructs generated by N-/C-terminal polar deletion, non-tagged secretagogin was co-expressed with His_6-tagged SNAP-25c/syntaxin-4Hb constructs in 2 ml LB cultures using the above expression methods. Interactions were tested as above. Each buffer was supplemented with 2 mM $CaCl_2$ to retain Ca^{2+}-dependent protein interactions during cell lysis and Ni-NTA-binding. Cell lysates, extracted proteins (soluble fraction), flow-through (unbound proteins) and eluted fractions (His-tagged target proteins and non-tagged secretagogin if bound to the target) were collected and analyzed by SDS-PAGE.

For testing mouse secretagogin binding to H_{abc}-domain constructs of syntaxin-4, samples containing purified TEV-cleaved (non-tagged) secretagogin and His_6-tagged target proteins were prepared at 100 μM in binding buffer (25 mM Tris-HCl and 300 mM NaCl at pH 8.0) supplemented with 10 mM imidazole and 2 mM $CaCl_2$. Samples were incubated at 21°C for 30 min to allow binding. Subsequently, samples were incubated with pre-washed Ni-NTA beads at 4°C for 45 min. His-tagged proteins or their protein complexes with non-tagged secretagogin were purified as above using buffers containing 2 mM $CaCl_2$ throughout. Flow-through and eluted fractions were collected and analyzed by SDS-PAGE.

Differential scanning fluorimetry

Thermal stability of mouse and human secretagogin and their complexes was monitored by following their thermal denaturation kinetics (3) using SYPRO Orange™ (Life Technologies). Recombinant mouse secretagogin and its respective target proteins (or their fragments) were dispensed in 96-well PCR plates (Bio-Rad) at 10 μM and 50 μM concentration, respectively, in 25 mM Tris-HCl, 150 mM NaCl buffer (pH 7.5) with/without 2 mM $CaCl_2$ or 10 mM EDTA. Individual proteins served as controls. Subsequently, SYPRO Orange was added at 5,000x dilution to each well, plates were covered with optical sealing tape (Bio-Rad), and shaken at 1,000 rpm for 2 min to remove air bubbles. Fluorescence intensity was monitored in a CFX-96 real-time PCR (Bio-Rad) using excitation at 470 nm and emission at 570 nm during increasing the temperature from 20 °C to 95 °C with a ramping rate of 1.0 °C/min and recording data points at every 0.2 °C per step. Fluorescence intensity data were processed using the CFX manager software (Bio-Rad).

Isothermal titration calorimetry

The standard buffer of the protein samples was first exchanged to 25 mM HEPES (pH 8.0), and 150 mM NaCl using NAP-5 columns (Cytiva). Recombinant mouse or human secretagogin and their target proteins were prepared at 10-100 and 80-1500 μM concentrations (for details, see Table *S2*), respectively, in the presence of 2 mM $CaCl_2$ or 10 mM EDTA. Titration was done on a MicroCal iTC_{200} device (GE Healthcare) by 16 injections of 1.0–2.0 μl of concentrated target protein solution (initial concentration 80-1,500 μM) to the cell containing 300 μl of the protein solution (10-100 μM). Dilution controls of the proteins tested were done in the same manner but with buffer only. Reactions were at 20 °C with an equilibration time of 90-120 s between injections. Data were analyzed and fitted to a single-site binding model using the MicroCal iTC_{200} software (GE Healthcare).

Mass photometry

Mass photometry (4,5) was carried out using a Two MP mass photometer (Refeyn) on 15H microscopy coverslips that were cleaned with isopropanol and water at 22 °C. Samples of mouse secretagogin and monomeric mouse syntaxin-4 comprising the SNARE-helix and the H_{abc} domain (lacking the N-peptide) were freshly prepared by SEC at 12 μM concentration (Fig. *S10*). Protein samples were kept in 25 mM Tris-HCl, 150 mM NaCl, 2 mM $CaCl_2$ (pH 8.0), and applied onto the coverslips. Proteins were tested alone or in mixtures at a final concentration of 25-30 nM. A sample of the original, high concentration (100 μM) syntaxin-4 construct that contained both oligomeric and monomeric forms was also tested. Data from the 2.87 μm × 10.80 μm instrument field of view were collected for 60 s at a frame rate of 1 kHz with frame binning of 10. Data acquisition was carried out with AcquireMP v2.4.0 (Refeyn). Data were analyzed in DiscoverMP v2.4.0 (Refeyn). Scattering intensities were plotted.

Protein crystallization and structure determination

Crystals of the mouse secretagogin/GFP-SNAP-25 complex were produced at 4 °C using the vapor diffusion method. Crystallization drops were prepared by the MOSQUITO crystallization robot (TTP LabTech) in CORNING 3350 plates (Sigma-Aldrich) by mixing 0.2 μL of the protein solution at 24.8 mg/mL (in 25 mM Tris-HCl (pH 8,0), 150 mM NaCl, 2 mM $CaCl_2$) with 0.1 μL of the well solution (0.1 M malic acid/imidazol/borate buffer (MIB, pH 6.0), 25% PEG1500). Crystals for data collection were picked in a crystal mounting loop (Mitigen), cryo-protected by dipping them in mother liquor with 30% PEG1500, flash frozen, and stored in liquid N_2.

Crystals of the human secretagogin/GFP-SNAP-25 complex were also produced at 4 °C using the vapor diffusion method. Crystallization drops were prepared by the MOSQUITO crystallization robot (TTP LabTech) in CORNING 3350 plates (Sigma-Aldrich) by mixing 0.2 μL of the protein solution (15.4 mg/mL, in 25 mM Tris-HCl (pH

8,0), 150 mM NaCl, 2 mM $CaCl_2$) with 0.1 µL of the well solution (0.2 M Na-acetate, 20% PEG3350). Crystals for data collection were picked in a crystal mounting loop (Mitigen), cryo-protected by dipping them in mother liquor with 30% PEG1500, flash frozen, and stored in liquid N_2.

Crystals of the mouse secretagogin/GFP-syntaxin-4 complex were produced at 4 °C using the vapor diffusion method, too. Crystallization drops were prepared by the MOSQUITO crystallization robot (TTP LabTech) in CORNING 3350 plates (Sigma-Aldrich), by mixing 0.15 µL of the protein solution (21.9 mg/mL, in 25 mM Tris-HCl (pH 8.0), 150 mM NaCl, 2 mM $CaCl_2$, 2 mM DTT) with 0.1 µL of the well solution (0.1 M Na-cacodylate (pH 5.5), 0.2 M NH_4SO_4, 25% v/v PEG smear medium, Molecular dimensions, MD2-100-259). Crystals for data collection were picked in a crystal mounting loop (Mitigen) without cryo-protection, flash frozen, and stored in liquid nitrogen.

X-ray diffraction datasets for mouse secretagogin/GFP-SNAP-25 and for human secretagogin/GFP-SNAP-25 complexes were collected at 100 K at the BIOMAX beamline of the MAX-IV synchrotron (Lund, Sweden) at 2.35 Å (X-ray λ = 0.97625 Å) and 2.30 Å (X-ray λ = 0.97996 Å) resolution, respectively. X-ray diffraction datasets for mouse secretagogin/GFP-syntaxin-4 were collected at the European Synchrotron Radiation Facility (Grenoble, France) beamline ID30B at 100 K (X-ray λ = 0.968626 Å) at 2.65 Å resolution. Crystal characterization and data collection parameters were based on MxCUBE (6) or EDNA (7). Diffraction data were indexed and integrated using XDS (8) and scaled by AIMLESS from the CCP4i suite (9). X-ray diffraction data statistics were shown in Table *S3*.

The structures of both the mouse secretagogin/GFP-SNAP-25 and the human secretagogin/GFP-peptide complexes were solved by molecular replacement using MOLREP (10) and PHASER (11) in the space group $P2_12_12_1$ using the coordinates of the crystallization chaperon GFP (PDB:1EMA, 12), as well as the structure of the ligand- and Ca^{2+}-free zebrafish (*Danio rerio*) secretagogin (PDB:2BE4, 13). After locating the two GFP molecules, individual EF-hand domains and half EF-hand modules were used in consecutive PHASER runs.

The structure of the mouse secretagogin/GFP-syntaxin-4-derived peptide complex was solved by molecular replacement using PHASER (11) in the space group $P2_12_12_1$ with the coordinates of the crystallization chaperon GFP, and the structure of the ligand- and Ca^{2+}-free mouse secretagogin B and C domains being used as search models. The solutions were confirmed by visual inspection of the electron density maps after refinement.

The models were completed by manual model building in COOT (14) interspersed by crystallographic refinement by REFMAC-5 (15) or PHENIX (16). Structural models were validated using COOT and MOLPROBITY (17). The model of the mouse secretagogin/GFP-SNAP-25-derived peptide complex contains two copies of the protein complex formed between mouse secretagogin and GFP-SNAP-25, 8 Ca^{2+} ions, 4 bound at each of the secretagogin molecules and 369 waters. The crystallographic refinement statistics resulted in R_{cryst}/R_{free} values of 0.194/0.248, with 963 residues (99.6%) in the allowed and 4 outliers (0.4%) according to their Ramachandran plot. The structure of the human secretagogin/GFP-SNAP-25-derived peptide complex crystallized with the same packing and lattice harboring two complexes, 8 Ca^{2+} ions, 2 acetate ions, one TRIS molecule and 422 crystallographic water molecules. Refinement statistics resulted in R_{cryst}/R_{free} values of 0.188/0.261, with 981 residues (100%) in the allowed and no outliers according to a Ramachandran plot. The model of the mouse secretagogin/GFP-syntaxin-4-derived peptide complex contains 2 copies of the assembly, 8 Ca^{2+} ions, 1 cacodylate ion, and 12 crystallographic water molecules. Refinement statistics resulted in R_{cryst}/R_{free} values of 0.213/0.268, with 837 residues (99.8%) in the allowed and 2 outliers (0.2%) according to aa Ramachandran plot. Crystal contacts were analyzed using the PISA algorithm (18) and figures were made in PyMOL (www.pymol.org). Analysis of the residue conservation in 3D was based on the CONSURF algorithm (19,20) utilizing 24 vertebrate sequences and the coordinates of the secretagogin protein solved in this work. Refinement statistics and model parameters are shown in Table *S3*. Crystallographic data were deposited at the Protein Data Bank under accession codes 8BAN, 8BAV, 8BBJ.

Immunohistochemistry

Mice (C57Bl6/J, 12-week-old, *n* = 3) were kept under standard housing conditions (12/12 light/dark cycle) with food and water available *ad lib*. The Austrian Federal Ministry of Education, Science and Research granted approval for this study (2021-0.721.018). Experimental procedures conformed to the 2010/63 European Communities Council Directive. Mice were deeply anesthetized by isoflurane (5% with 1 L/min flow rate of tubed air) and organs were perfusion-fixed by transcardially applying 4% (wt/vol) paraformaldehyde in 0.1 M phosphate buffer (PB). Dissected pancreata were post-fixed by immersion in the same fixative at 4 °C overnight, and then cryoprotected in 30% sucrose in PB at 4 °C for 3 days. Ten-µm-thick sections were cut onto fluorescence-free glass slides on a cryostat microtome.

The sections were washed multiple times in 0.1 M PB. Non-specific immunoreactivity was reduced by incubating the sections in a mixture of 5% normal donkey serum (NDS; Jackson ImmunoResearch) and 0.3% Triton X-100 (Sigma) in 0.1 M PB at 22-24 ºC for 2 h. Sections were then exposed (at 4 °C for 24h) to specific combinations of primary antibodies (secretagogin: Synaptic System, #436004, guinea pig, 1:1000; SNAP-25: Synaptic System, 111002, rabbit, 1:500; syntaxin-4: Chemicon, AB5330, rabbit, 1:500; glucagon: Sigma, G2654, mouse, 1:2,000) diluted in 0.1 M PB to which 0.1% NDS and 0.3% Triton X-100 has been added. After multiple washing steps in 0.1 M PB, immunoreactivities were revealed by carbocyanine (Cy) 2-, 3 or 5-tagged secondary antibodies raised in donkey (1:500 (Jackson ImmunoResearch), at 22–24 °C for 2 hrs. Hoechst 33,342 (1:10,000; Sigma) was routinely used as nuclear counterstain. Glass-mounted sections were coverslipped with Entellan (Sigma) in toluene as solvent.

Sections were inspected and images acquired on a LSM880 confocal laser-scanning microscope (Zeiss) at either 20x or 63x primary magnification with the pinhole set to 0.5 – 0.7 µm. Emission spectra for each dye were limited as follows: Cy2 (505–540 nm), Cy3 (555–610 nm) and Cy5 (650-720 nm). Super-resolution images were made by using an AiryScan detector module (Zeiss). Multi-panel figures were assembled in CorelDraw 2024 (Corel Corp.).

Cell culture

Rat insulinoma cells (INS-1E) that resemble β-cells were kept in RPMI 1640 medium (Gibco) containing 5% fetal bovine serum, penicillin (100 U/ml), streptomycin (100 µg/ml), sodium pyruvate (1 mM) and 2-mercaptoethanol (50 µM; all from Gibco). Cells were maintained in a CO_2 incubator (Thermo Scientific) under normal conditions (5% CO_2, 37°C, 95% humidity) with the medium replaced every other day.

For overexpression experiments, INS-1E cells were transfected with 1 µg expression vector (pcDNA-3.1, pcDNA-mSTX4) using a Nucleofector system (Lonza), and plated at a density of 1 million cells/well in 6-well plates coated with poly-D-lysine (Sigma). For other experiments, 12-well pre-coated plates were used at a seeding density of 200,000 cells/well.

To measure insulin secretion, INS-1E cells were first treated with H_2O_2 (100 µM or 200 µM, Sigma) for 12 h or stimulated by 90 mM KCl in Krebs-buffer with/without 1 µM phorbol 12,13-dibutyrate (PDBu) for 30 min. Supernatants were collected and analyzed with an ultrasensitive insulin ELISA kit (Mercodia) according to the manufacturer's instructions. RNA from cell pellets was isolated by an RNA mini kit (BioRad), and its concentration normalized once measured on a Nanodrop. cDNA was reverse-transcribed using a high-capacity cDNA reverse transcription kit (ThermoFisher). PCR reactions were performed using the following primer pairs:

Target mRNA	Forward primer	Reverse primer
Gapdh	5´-CAA GTT CAA CGG CAC AGT CA-3´	5´-CCC CAT TTG ATG TTA GCG GG-3´
Scgn	5´-CCC AGA AGT GGA TGG ATT TG-3´	5´-GTT GGG GAT CAG GGG TTT AT-3´
Snap25	5´-CAT GCT GCA GCT GGT CGA AG-3´	5´-TCG GCC TCC TTC ATG TCT TGG-3´
Stx4	5´-AGA GAA GAA CGT GGA GCG CA-3´	5´-CGG GTC ACC TGT GTG TCC TT-3´
Xbp1s	5´-GAG TCC GCA GCA GGT G-3´	5´-GTG TCA GAG TCC ATG GGA-3´

Statistics

Data were analyzed and visualized using the GraphPad software. A p value of < 0.05 was considered statistically significant. Data were evaluated using Student's t-test or one-way ANOVA, as appropriate. Data were expressed as means ± s.e.m.

Supporting References

1. Oke M, Carter LG, Johnson KA, et al. The Scottish Structural Proteomics Facility: targets, methods and outputs. *J Struct Funct Genomics*. 11, 167-80 (2010).
2. Vander Kooi CW. Megaprimer method for mutagenesis of DNA. *Methods Enzymol*. 529, 259-69 (2013).
3. Reinhard L, Mayerhofer H, Geerlof A, Mueller-Dieckmann J, Weiss MS. Optimization of protein buffer cocktails using Thermofluor. *Acta Crystallogr Sect F Struct Biol Cryst Commun*. 69, 209-214 (2013).
4. Young G, Hundt N, Cole D, Fineberg A, Andrecka J, Tyler A, Olerinyova A, Ansari A, Marklund EG, Collier MP, Chandler SA, Tkachenko O, Allen J, Crispin M, Billington N, Takagi Y, Sellers JR, Eichmann C, Selenko P, Frey L, Riek R, Galpin MR, Struwe WB, Benesch JLP, Kukura P. Quantitative mass imaging of single biological macromolecules. *Science.* 360, 423-427 (2018).
5. Wu D, Piszczek G. Measuring the affinity of protein-protein interactions on a single-molecule level by mass photometry. *Anal Biochem*. 592, 113575 (2020).
6. Mueller, U.; Thunnissen, M.; Nan, J.; Eguiraun, M.; Bolmsten, F.; Milàn-Otero, A.; Guijarro, M.; Oscarsson, M.; de Sanctis, D.; Leonard, G. MXCuBE3: A New Era of MX-Beamline Control Begins. *Synchrotron Radiat. News* 30, 22–27 (2017).
7. Incardona, M. F.; Bourenkov, G. P.; Levik, K.; Pieritz, R. A.; Popov, A. N.; Svensson, O. EDNA: A Framework for Plugin-Based Applications Applied to X-Ray Experiment Online Data Analysis. *J. Synchrotron Radiat.* 16, 872–879 (2009).
8. Kabsch W. XDS. *Acta Crystallogr D Biol Crystallogr.* 66, 125-132 (2010).
9. Winn MD, Ballard CC, Cowtan KD, Dodson EJ, Emsley P, Evans PR, et al. Overview of the CCP4 suite and current developments. *Acta Crystallogr D Biol Crystallogr*. 67, 235-242 (2011).
10. Vagin A, Teplyakov A. Molecular replacement with MOLREP. *Acta Crystallogr D Biol Crystallogr*. 66, 22-25 (2010).
11. A.J. McCoy, R.W. Grosse-Kunstleve, P.D. Adams, M.D. Winn, L.C. Storoni & R.J. Read. Phaser Crystallographic Software. *J. Appl. Cryst.* 40, 658-674 (2007).
12. Ormö M, Cubitt AB, Kallio K, Gross LA, Tsien RY, Remington SJ. Crystal structure of the Aequorea victoria green fluorescent protein. *Science* 273, 1392-5 (1996).
13. Bitto E, Bingman CA, Bittova L, Frederick RO, Fox BG, Phillips GN Jr. X-ray structure of Danio rerio secretagogin: A hexa-EF-hand calcium sensor. *Proteins*. 76, 477-83 (2009).
14. Emsley P, Lohkamp B, Scott WG, Cowtan K. Features and development of Coot. *Acta Crystallogr D Biol Crystallogr*. 66, 486-501 (2010).
15. Murshudov GN, Skubák P, Lebedev AA, Pannu NS, Steiner RA, Nicholls RA, et al. REFMAC5 for the refinement of macromolecular crystal structures. *Acta Crystallogr D Biol Crystallogr*. 67, 355-367 (2011).
16. Adams, P. D.; Afonine, P. V.; Bunkóczi, G.; Chen, V. B.; Davis, I. W.; Echols, N.; Headd, J. J.; Hung, L. W.; Kapral, G. J.; Grosse-Kunstleve, R. W.; McCoy, A. J.; Moriarty, N. W.; Oeffner, R.; Read, R. J.; Richardson, D. C.; Richardson, J. S.; Terwilliger, T. C.; Zwart, P. H. PHENIX: A Comprehensive Python-Based System for Macromolecular Structure Solution. *Acta Crystallogr. Sect. D Biol. Crystallogr*. 66, 213–221 (2010).
17. Williams CJ, Hintze BJ, Headd JJ, Moriarty NW, Chen VB, Jain S, et al. MolProbity: more and better reference data for improved all-atom structure validation. *Protein Science* 27, 293-315 (2018).
18. Krissinel E, Henrick K. Inference of macromolecular assemblies from crystalline state. *J Mol Biol*. 372, 774-797 (2007).
19. Landau M., Mayrose I., Rosenberg Y., Glaser F., Martz E., Pupko T. and Ben-Tal N. ConSurf 2005: the projection of evolutionary conservation scores of residues on protein structures. *Nucl. Acids Res.* 33, W299-W302 (2005).
20. Ashkenazy H., Abadi S., Martz E., Chay O., Mayrose I., Pupko T., and Ben-Tal N. ConSurf 2016: an improved methodology to estimate and visualize evolutionary conservation in macromolecules. *Nucl. Acids Res.* 44, W344-50 (2016).
